# Supplementary material for: A stochastic model for simulating ribosome kinetics in vivo
Source: PLoS Comput Biol. 2020 Feb 12;16(2):e1007618. doi: 10.1371/journal.pcbi.1007618 (PMC7015319; doi:10.1371/journal.pcbi.1007618)
Supplement: S1 Text — (PDF) [file pcbi.1007618.s001.pdf]

A Stochastic Model of Prokaryote Ribosome Kinetics

Translation of an mRNA by a bacterial ribosome can essentially be broken up into three stages: (1) formation of the 30S pre-initiation complex (30S:PIC) followed by ribosomal initiation at a start codon within a ribosomal binding site (RBS), (2) elongation and protein synthesis, and (3) termination at a stop codon and recycling of the ribosomal machinery. Each of these three stages encompasses a set of biochemical reactions, which may involve additional host protein factors such as Ef-G. Moreover *in vivo*, there are additional housekeeping reactions, such as aminoacylation of tRNAs, Ef-Ts mediated exchange of GDP for GTP on Ef-Tu, GTP production, etc. which are occurring in the background during ribosomal translation on the mRNAs. These additional cellular reactions are required for protein synthesis to proceed uninterrupted. Each of the stages of the ribosome translation of an mRNA and the additional cellular reactions required to maintain protein production are detailed below, along with a discussion of the experimental measurements and choices of kinetic rates used in the model. In addition, I discuss estimates for total concentrations of different cellular factors important for ribosomal function *in vivo*, such as elongation factors and initiation factors, and the subsequent tuning of kinetic parameters so that the model reproduces observed features such as the rate of amino acid incorporation by *E. Coli* ribosomes and the expected spacing of ribosomes on mRNAs for different growth rates.

Model for 30S:PIC Formation. In bacterial systems, the ribosome consists of 30S and 50S subunits. The smaller 30S subunit first interacts with three additional host proteins called initation factors (IF1,IF2,IF3) and the formylated methoinine tRNA (fMet-tRNA) to construct a pre-initiation complex (30S:PIC). Once formed, the 30S:PIC can subsequently interact with the ribosomal binding sites (RBS) present in mRNAs to initate protein synthesis. Milon *et al.* (1) performed a detailed kinetic analysis of the assembly landscape for the 30S:PIC using FRET fluorescence binding assays. The conclusions of this study were that while the IFs and fMet-tRNA can bind the 30S subunit mostly independently of each other, there was a favoured kinetic pathway of formation in which IF3 and IF2 join first, followed by IF1 and fMet-tRNA. There are multiple pathways of formation of the 30S:PIC, and Milon *et al.* have noted that it is possible for IFs to join the 30S subunit after the 30S subunit binds to an RBS within an mRNA. However, I have simplified the model in this work to the single dominant kinetic pathway discussed in Milon (1), with 30S:PIC formation required to preceed mRNA binding. Kinetic modeling of the initiation process in this work has shown that IFs binding to free 30S subunits occurs quickly ($< 300ms$) and in far less time than would be required for the RBS site to clear from the previous ribosomal initiation event ($> 1s$), suggesting that in the quasi-steady state the majority of 30S subunits have formed the 30S:PIC. The pathway is illustrated in Figure A, while Table A lists the kinetic parameters that I have used in the model that were obtained from the fluorescence binding assays in Milon (1).

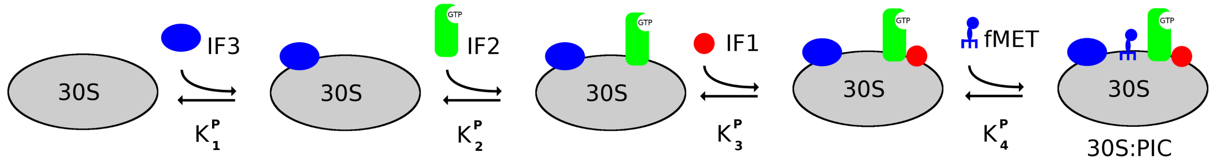

Figure A. Kinetic model for 30S:PIC formation. The dominant kinetic pathway determined from FRET fluorescence assays (1) involves the initial binding of IF3, followed by the subsequent binding by the GTP-bound IF2, IF1, and finally fMet-tRNA. Kinetic parameters measured in (1) are listed in Table A.

Model of 30S:PIC binding to mRNA. During initiation of the ribosomal translational machinery on an mRNA, the 30S:PIC binds to a RBS to form the 30S initiation complex (30S:IC) and then undergoes subsequent maturation to become a 70S elongation complex (70S:EC) after completing a series of kinetic checkpoints that ensure proper identification of, and initiation on, the correct start codon. The 30S:PIC and 30S subunit tend to have a preference for binding large stretches of single-stranded nucleotides (≈ 40 nt), although it has been shown to also be capable of binding to weakly structured areas containing a few hairpins (2, 5). Moreover in bacteria, access to the RBS tends to be regulated by mRNA secondary structure, which can sequester the start codon in base-paired regions of the mRNA, preventing the recognition of the start codon by the 30S:PIC. Such structured areas in RBSs are known to exist in the RNA bacteriophages MS2 and Qβ (15) and Van Duin has shown that translation initiation would be extermely poor/slow if the ribosome was forced to wait until the RNA structure around the coat gene in MS2 first unfolded (2). Thus it has been postulated that standby sites, *i.e.* areas of weak secondary structure that the 30S:PIC is capable of binding to, allow the 30S:PIC to wait on the mRNA until unfolding occurs (2). I have incorportated this feature into the model along with a standard binding pathway to unstructured mRNA as shown in Figure B. The binding pathway is equivalent to that of Van Duin's (2), with an additional recognition step with kinetic rate K_3^B that corresponds to the third kinetic checkpoint in (1). On and off rates of the 30S:PIC binding to mRNA used in the model are given in Table B. These were estimated for the unstructured pathway using 30S:PIC unbinding to poly-U RNA (5). For the off rate from the standby site, I have used a value such that the K_d of 30S:PIC binding is 100-fold higher than that for the binding of 30S:PIC to unstructured RNA, as was done in van Duin's model of the standby site (2). I model the recognition step as the energy of interaction of any potential Shine-Delgarmo (SD) sequence in the mRNA with the complementary anti-SD sequence in the 16S rRNA. Thus, the rates K_3^B, K_{-3}^B, K_F, K_{-F}, and K_{SF}, K_{-SF} will depend on the mRNA sequence and/or secondary structure and can be determined from the Turner nearest neighbour model of base-pair stacking (14) and the lowest energy barrier

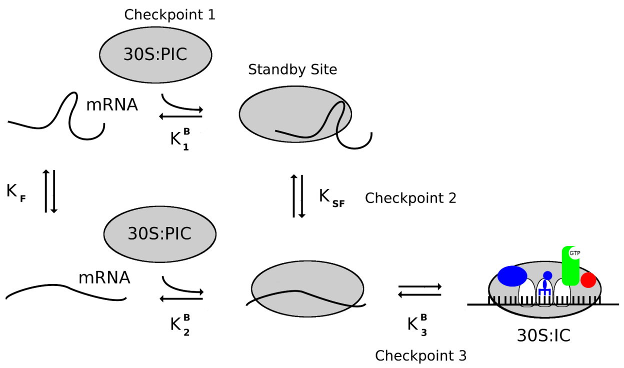

Figure B. Kinetic model of 30S:PIC binding to mRNA. The model includes two pathways to formation of the 30S:IC: (1) the standby pathway, where the 30S:PIC first binds to a weakly structured area of the mRNA and waits until RNA unfolding presents the start codon, and (2) a pathway in which the 30S:PIC binds to unstructured RNA. Kinetic parameters used in the model, measured by Studer and Joseph (5), are listed in Table B.

pathway between the folded and unfolded state using a path-dependant computational method such as the Morgan-Higgs model (47).

Model of 70S Initiation. After binding of the 30S:PIC to mRNA and formation of the 30S:IC by interaction of the 16S rRNA with the SD sequence and inital interactions of the fMet-tRNA with the mRNA, the 30S:IC undergoes a futher set of kinetic checkpoints to ensure initation on the correct start codon (6–9). Following 30S:IC formation, the 50S subunit joins the 30S:IC and promotes rapid IF2-dependant GTP hydrolysis. After hydrolysis, the 50S subunit then can either dissociate reversably or form a more stable complex (70S:IC), which involves a conformational change to the 50S subunit and movement of the fMet-tRNA into the P-site (6, 9) followed by Pi release. Finally, IF1 and IF2 are released followed by IF3 ejection and maturation of the 70S:IC into the elongation complex. There is some discrepancy between the order of fMet-tRNA accomodation into the P-site, with Grigoriadou *et al.* reporting that it occurs prior to Pi (6) release, while Goyal *et al* reporting that it occurs thereafter (9). I have modeled the movement of fMet-tRNA into the P-site as occuring prior to Pi release, as this would be the last point for the 30S:IC to dissociate reversably in the model before being commited to maturation to the 70S:EC. Finally, it should be further noted that Grigoriadou *et al.* reported that IF2 can interact with both 50S and 30S subunits (6). However, I have neglected interaction of IF2 with 50S subunits as the interaction with 50S is about $K_d = .4\mu M$, at least 10 times weaker then interaction with the 30S subunit. Figure C illustrates the kinetic pathway and kinetic rates, measured from fluorescence binding assays (6, 9), as given in Tables B and C.

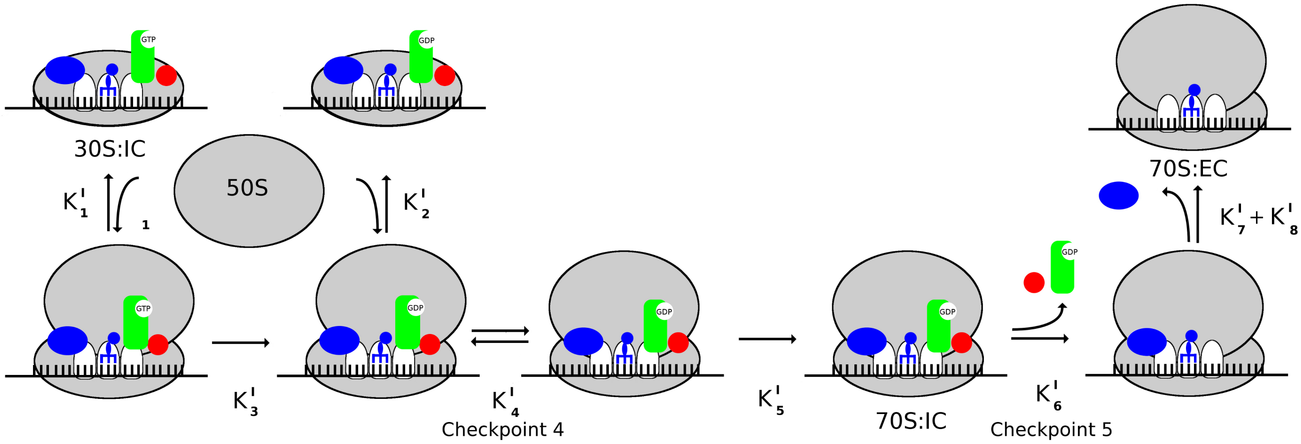

Figure C. Kinetic model of 70S Initiation. Kinetic parameters used in the model were measured by fluorescence binding assays (6, 9) and are listed in Tables B and C.

Dependence of Initiation Efficiency on the Kinetic Checkpoints. Here I describe how the initiation efficency on an mRNA can be theoretically taken into account in the model based on its explicit nucleotide sequence. In total, there are 5 kinetic checkpoints (1, 10) in which the sequence and structure of the mRNA can alter the kinetics of initiation, thereby affecting the translational initiation efficiency on a given mRNA sequence. Previously, Salis (3) had reported on an estimate of the translational initiation rate based on mRNA structure unfolding and the energetics of interaction with an SD site in the mRNA. The Salis model estimates the total initiation rate from $r \propto e^{\beta\Delta G_{tot}}$. Here in this work, the initiation rate is predicted from the time that it takes for the 30S:PIC to pass through the following kinetic checkpoints that will depend on the mRNA sequence

and its structure: (1) the initial binding of the 30S:PIC to mRNA, which depends on the secondary structure around the start codon and the presence of a standby site - K_1^B or K_2^B, (2) mRNA unfolding - K_F or K_{SF}, (3) recognition of the SD sequence by 16s rRNA - K_3^B, (4) 50S subunit binding - K_1^I or K_2^I and movement of the tRNA into the P-site and codon recognition - K_4^I, and (5) ejection of IFs.

The kinetic rates for RNA folding at the second kinetic checkpoint are computed from:

$$\begin{aligned} \frac{K_F}{K_{-F}} &= e^{-\beta \Delta G_F} && [1] \\ K_F &= A e^{-\beta \Delta G^{\dagger}} && [2] \\ K_{SF} &= K_F && [3] \\ K_{-SF} &= 100 K_{-F}, && [4] \\ & && [5] \end{aligned}$$

where the factor $A = 3 \times 10^7 s^{-1}$ is the attempt frequency (2) and $\Delta G^{\dagger}$ is the energetic barrier to unfolding, while K_{-SF} is fixed from the first law of thermodynamics in the kinetic reaction network shown in Figure B. Here, ΔG_F, the total change in energy due to mRNA unfolding of the translation initiation region (TIR), is approximately equivalent to $\Delta G_{mRNA} + \Delta G_{standby}$ in Salis. I calculate ΔG_F and $\Delta G^{\dagger}$ using the Morgan-Higgs greedy algorithm (47), which identifies an optimal kinetic pathway between two RNA structures, and use the Turner 99 nearest neighbour parameters for base-pair stacking (14) to calculate the transition energies for the optimal unfolding pathway.

The SD/anti-SD recognition step with kinetic rates

$$\frac{K_3^B}{K_{-3}^B} = e^{-\beta \Delta G_{sd}} \qquad [6]$$

comprise the third kinetic checkpoint, where ΔG_{sd} is the energy change due to the interaction of the anti-SD sequence in the 16S rRNA with the SD sequence in the mRNA. These rates and their equivalent energy barrier correspond to $\Delta G_{mRNA:RNA}$ in Salis. It is difficult to estimate a value for the SD/anti-SD on rate K_3^B as there is limited experimental information in the literature on the individual kinetic steps that take place during the initial ribosome binding to the mRNA. I have found that a rate of $K_3^B = 10^6$ gives reasonable estimates for the overall 30SPIC binding rate to the mRNA in both the standby and unstructured scenario that are consistent with Van Duin and colleague's work on the MS2 coat protein RBS (2).

The movement of the fMet-tRNA into the P-Site, kinetic checkpoint 4, is determined in the model according to

$$\frac{K_4^I}{K_{-4}^I} = e^{-\beta \Delta G_{cdn}}, \qquad [7]$$

where ΔG_{cdn} contains the energy of interaction of the fMet-tRNA anti-codon with the start codon ΔG_{start}, along with the energetic cost of non-optimal spacing between the SD site and the start codon, $\Delta G_{spacing}$. The interaction with the start codon ΔG_{start} can be obtained from Turner's nearest neighbour parameters and is roughly -1.2 kcal/mol for AUG start codons. For $\Delta G_{spacing}$, I use the formula from Salis (3),

$$\Delta G_{spacing} = \begin{cases} 0.048(s-5)^2 + 0.24(s-5) & s \geq 5 \\ 12.2\left(1 + e^{2.5(s-3)}\right)^{-3} & s < 5 \end{cases}, \qquad [8]$$

where s is the number of nucleotides between the 3' end of the SD sequence and the first nucleotide in the start codon. For an ideally spaced SD sequence and canonical AUG start codon, $s = 5$ and $\Delta G_{spacing} = 0$. Assuming that the kinetic rates determined from experiment correspond to the optimal spacing scenario, it can be seen that

$$\frac{K_4^I}{K_{-4}^I} = \frac{24}{2.1} = 11.43, \qquad [9]$$

giving $\Delta G_{cdn} = \Delta G_{start} = -1.50$ kcal/mol, which is roughly near the value expected from the Turner nearest neighbour model for AUG start codons. Sequence dependant changes to ΔG_{start} and $\Delta G_{spacing}$ will therefore have an impact on the rates K_4^I and K_{-4}^I in the model and the efficiency of the ribosome clearing kinetic checkpoint 4.

The model currently only considers 70S initations at canonical AUG start codons. However, it does have the capability of incorporating noncanonical start codons (*e.g.*, GUG, UUG, CUG, AUU, AUA) via adjustments to the rates of the five kinetic checkpoints. Hecht *et al.* have performed a measurement of translational initation frequency from all 64 start codons showing reasonable efficiency at GUG and UUG codons (11) and Milon *et al.* have observed that after closing of the 50S subunit to the 70S:IC, IF3 ejection proceeds 10-100 times slower on noncanonical start codons (7), which corresponds to the fifth kinetic checkpoint. These experimental observations can be used to test and tune the models predictability of protein synthesis rates at noncanonical starts in future.

Finally, it should be noted that the maximal loading rate of ribosomes on the mRNA occurs in the model when there is limited secondary structure sequestering a canonical start codon and there is optimal spacing between the start codon and any SD sequence. In this scenario, the spacing between subsequent ribosomes on the mRNA will be limited by the time required for

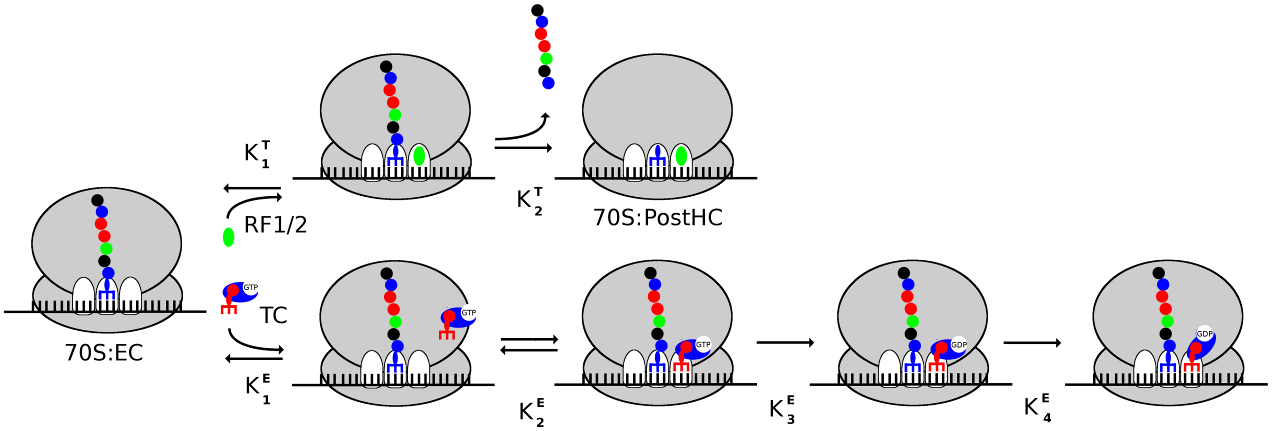

Figure D. Kinetic model of 70S Elongation. The states of the ribosome during ternary complex selection and GTP hydrolysis by Ef-Tu or termination and peptide release are illustrated. Kinetic rates are listed in Tables D and E for the elongation reactions and Tables F and G for the termination reactions by RF1 and RF2, respectively.

the 70S:IC complex to mature into the 70S:EC as additional ribosomes can be loaded in a standby site upstream of the start codon to wait until the 70S:IC clears the RBS to begin further rounds of intitiation. Such a polysome loading feature has been noted by Rodnina and colleagues for highly expressed mRNA (24). The rates used in this study are for such a maximal loading situation, and therefore I expect changes to kinetic rates from such features as a lack of SD sequence or mRNA secondary structure, to increase this delay and result in lower protein expression. Future work will test and tune the kinetic parameters for such situations.

Model of 70S Elongation. After the kinetic steps involved with 70S initiation on the mRNA are completed, the ribosome enters the elongation phase of protein synthesis, denoted as 70S:EC. At this stage, ternary complex (TC), consisting of an aminoacyl-tRNA (aa-tRNA) in complex with the GTPase elongation factor Tu (Ef-Tu) and bound GTP, bind to the ribosome and transfer the amino acid to the growing peptide chain. Aminoacyl-tRNAs must be selected from the pool of available TC, checked for the correct anti-codon, then have the amino acid covalently linked to the C-terminal end of the elongating peptide. Figure D shows the states of the ribosome during aa-tRNA selection and GTP hydrolysis by Ef-Tu, while Figure E shows the amino acid accommodation / rejection and translocation phase of elongation.

The kinetic steps of aa-tRNA selection and incorporation are probably the most well understood of the overall elongation process and have been previously described by Pape (40) and Rodnina (41). Here, I replicate previous kinetic models of ribosome elongation and include the following steps: (1) ternary complex binding, (2) codon recognition, (3) GTP activation and hydrolysis, (4) Ef-Tu rearrangement, (5) aa-tRNA accommodation or (6) aa-tRNA rejection. The kinetic rates for these steps have been extensively measured by both Pape and Rodnina (40, 41) at both 20 and 37 degrees C. Additionally, the measurements made at 37 degrees C have been adjusted using a global fitting procedure to reproduce expected amino acid incorporation rates observed for different growth rates of *E. coli* (42). Tables D and E list the kinetic rates used in this work for both cognate and near-cognate tRNAs which can be used at any *E. coli* growth rate.

Codon/Anti-codon recognition in slightly more complex than simply an exact complementary match of the anti-codon to the codon sequence in the mRNA. For example, *E. coli* does not express all 61 tRNAs containing all possible anti-codons that could be encountered when decoding the 61 sense codons in the genetic code. Instead, *E. coli* expresses roughly 46 tRNAs and these have cognate recognition with multiple codons (16). For example, lysine is coded by two possible codons (AAA and AAG) but both codons can be recognised by a single tRNA with anti-codon UUU. Dong *et al.* lists the specific cognate codon recognition by each of the tRNAs (16) and I have incorporated this feature into the model. For near-cognate codon recognition I assume, as in Rudorf *et al.* (42), that tRNAs recognize a codon as a near-cognate one if the codon is one mutation away from a codon that the tRNA would recognise as cognate. The codon/anti-codon recognition is encoded in a 64×64 matrix in the computational code which allows identification of codon / anti-codon binding rate for any tRNA anti-codon / mRNA codon pair. Cognate or near-cognate rates are copied into this matrix based on the *E. coli* tRNA recognition table in Dong (16).

The translocation step of the elongation process is complex and involves binding of the GTPase elongation factor G (Ef-G) to the ribosome, where GTP hydrolysis drives translocation of the ribosome along the mRNA, movement of the tRNA from the A-site to the P-Site, and ejection of t-RNA from the E-site (45, 46). The translocation step, which follows GTP hydrolysis by Ef-G, likely involves a number of conformational rearrangements of the ribosome, specifically: ribosome unlocking, rearrangements of the 50S and 30S subunits, followed by relocking of the ribosome (13, 46). As many of the conformational rearrangements and their rates are unknown, I have modelled these as a single reaction step with the translocation event, as illustrated in Figure E. This kinetic model for translocation follows the one introduced by Ramakrishnan (13) and allows the translocation event to retain dependence on Ef-G and GTP concentration in the cell, without complicating the model

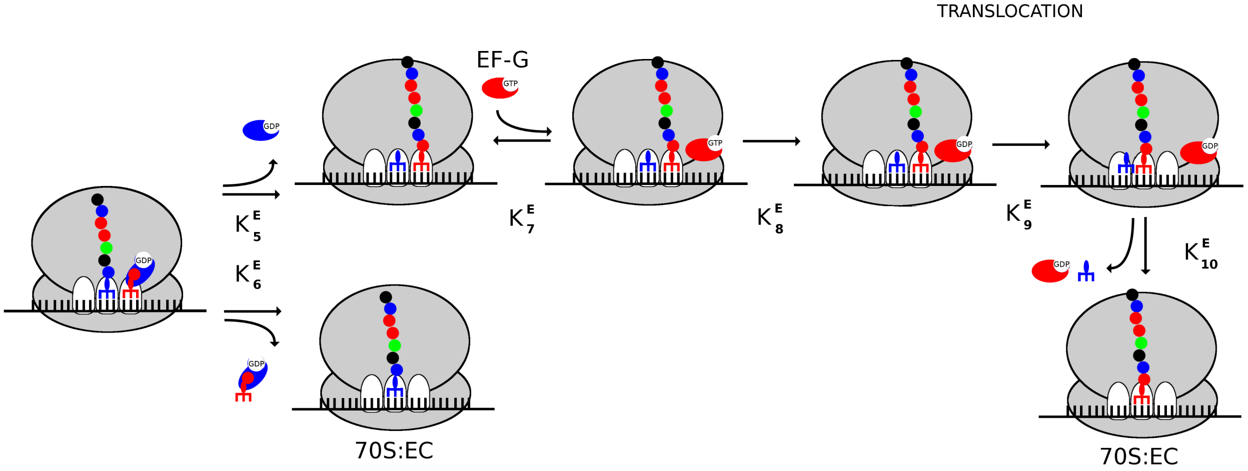

Figure E. Kinetic model of 70S Elongation. The states of the ribosome during aa-tRNA accommodation and Ef-G dependant translocation are illustrated. Kinetic rates are listed in Tables D and E for cognate and near-cognate codons, respectively.

with the specific rearrangement events that occur to the ribosome during translocation. Estimation of the kinetic rates at 37 degrees C have been done by both Katunin and Savelsberg (45, 46). Both Borg (12) and Rodnina (43) have noted that after GTP hydrolysis, tRNA movement on the ribosome is extremely rapid, and it is therefore difficult to measure a precise rate for translocation. This observation suggests that the translocation and Ef-G ejection rates require adjustment to fit with *in vivo* observations of amino acid incorporation (19). Supporting Tables D and E give the translocation kinetic rates used in the model for both near-cognate and cognate tRNAs. These rates give an average amino acid incorporation rate of 20-22 amino acids per second, consistent with elongation times at the highest bacterial growth rates. As discussed in the paper, these kinetic rates are consistent with the lower rates of amino acid incorporation (15-18 amino acids per second) seen at lower growth rates when the complex inter-dependence of free TC and free Ef-Ts is taken into account, negating the need for different kinetic parameters for different growth rates.

Model of 70S Termination and Protein Release. After the elongation phase, the ribosome encounters one of the three stop codons and then enters the termination and recycling phases. When a stop codon enters the A site of the ribosome in the pre peptide-hydrolysis complex (preHC), one of two class 1 release factors (RF1 or RF2) enters the A site on the ribosome and stimulates catalysis of the covalent bond between the C-terminal amino acid and the tRNA in the P site resulting in a post peptide-hydrolysis complex (postHC). Each release factor recognises two stop codons, with RF1 recognizing UAA or UAG and RF2 recognizing UAA or UGA (28, 33). There are two steps in the kinetic model of termination, RF1 or RF2 binding, and peptide release. I assume that RF1 and RF2 have the potential to terminate translation on sense codons that are one mutation away from the stop codons that they can recognise, consistent with Freistroffer *et al.* (28). Estimation of kinetic parameters for the peptide release process may be more complicated then other kinetic measurements, since some reactions seem very senstive to the chemical conditions of the experimental buffers. For example, Indrisiunaite *et al.* have noted that peptide hydrolysis is pH dependant, and that traditional experiments done in Tris-buffer have lower peptide release rates (36). Specifically, they have shown that a peptide release rate of $23s^{-1}$ can be expected *in vivo*. Finally, Hetrick *et al.* have measured binding rates for RF1 binding to UAA, CAA and UUU and CUC codons, finding rates between 15 and 32 $\mu M^{-1}s^{-1}$ (32). These measurements are consistent with measurements made by Zavialov *et al.* which estimated binding rates for RF1 and RF2 between 23 and 60 $\mu M^{-1}s^{-1}$ (30). Hetrick *et al.* have also measured off rates for RF1/RF2 unbinding from the preHC using RF1 and RF2 GAQ mutants, which are very slow at catalysing the peptide bond from the tRNA. They found off rates in the range of 0.0028 to 350 s^{-1} for RF1, depending on the codon in the A site of the preHC. Moreover, Adio *et al.* (33) have also recently measured RF1/RF2 binding to the preHC complex and found off rates on the order of $0.1 - 1.0s^{-1}$, sligtly greater than that measured by Freistroffer *et al.* (28).

Given this information in the literature, I have rescaled the off rates for RF1 and RF2 unbinding from the preHC to be $0.1s^{-1}$ and $1.3s^{-1}$, respectively, which are consistent with measurements by Adio *et al.* (33) and the RF1/RF2 recycling steps in the next section. I have kept the binding rates of RF1 and RF2 to the preHC to be the same as calculated in Freistroffer *et al.* (28) and have adjusted the peptide hydrolysis rate for RF1 and RF2 on stop codons to be $23s^{-1}$, consistent with estimates from Indrisiunaite *et al.* (36). To determine the off-rates of RF1 and RF2 from near cognate sense codons, I have used the K_M and k_{cat} values reported by Freistroffer *et al.* (28). The overall result is a RF1/RF2 binding and peptide release rate of $0.1s$ and a termination rate at sense codons at a frequency of 1 per 2.8x10^5 codon reading events, which is 10 times less frequent than expected from experimental estimates (50). To fit with experimental observations, either the concentration of RF1/RF2 needs to be higher or the rates of peptide-hydrolysis at sense codons by RF1 or RF2 need to be increased. There may be some evidence that RF1 and RF2 are actually higher then expected. Using the mdoel, if one computes the rate of stop-codon

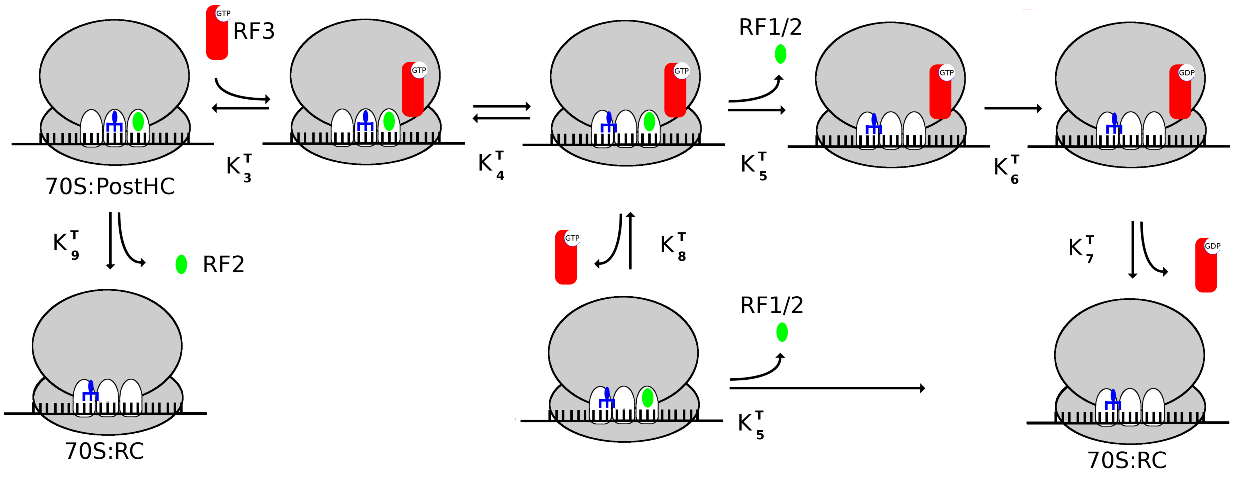

Figure F. Kinetic model of RF1/RF2 recycling. The kinetic reactions during recycling of the class 1 release factors (RF1/RF2) by class 2 release factor RF3 are shown. Kinetic rates are listed in Table H.

read-through due to competition between the ternary complex and RF1/RF2, the current rate of read through is about 3-9%, which is likely too high. Doubling RF1 and RF2 reduces the stop codon read through rate to roughly 2-5% and subsequently increases pre-mature termination to around 1 per 8x10^5 codon reading events. However, more experimental work needs to be done in order to determine which of these scenarios is correct. In summary, the termination and peptide release states are illustrated in Figure D and the kinetic rates I have used in the model are given in Table F for RF1 and Table G for RF2.

Model of 70S Recycling. After termination on a stop codon and release of the peptide, the GTPase class 2 release factor RF3 binds to the ribosome and stimulates release of the class 1 release factor bound to the ribosome (either RF1 or RF2). While the kinetic mechanisms of RF1/RF2 release from the ribosome post-HC and subsequent GTP hydrolysis by RF3 has been debated over the last decade providing conflicting models, a clearer picture of the kinetic mechanism has begun to emerge in the last few years. A number of studies in the early 2000's showed that RF3 binding to the ribosome accelerated release of RF1/RF2, and that release of RF1/RF2 regulated the GTPase activity of RF3 (29–31). This lead to a model of RF1/RF2 recycling that had RF1/RF2 release prior to RF3 release (31). After release of the class 1 factor, RF3 would undergo GDP/GTP exchange followed by GTP hydrolysis and release (31). Exchange of GDP for GTP was assumed to occur on RF3 bound to the ribosome, since it was shown that the affinity of free RF3 for GDP was much higher than for GTP, whereas on the ribosome this affinity preference switched (34). I have found several problems with this model, which suggest that an alternative model needs to be considered. First, because the concentrations of free RF3 are predicted to be quite low in the cell (approx. 431 per cell (27)) along with the observation that the GTP hydrolysis rate of RF3 is slow, i.e. $0.4 - 1.0s^{-1}$ (37, 38), I have found that the available free RF3 in the cell are unable to processes terminating ribosomes efficiently without causing substantial stalling of upstream elongating ribosomes on the mRNA if GTP hydrolysis is required to release RF1/RF2 every time. Second, the reported release rates of RF1/RF2 when in complex with RF3 on the ribosome of about $0.38s^{-1}$ (39), are too slow to processes terminating ribosomes efficiently, even when neglecting the hydrolysis time of RF3. Considering that at a high bacterial growth rate a gap of 46 nt (or center to center distance of roughly 88 nt) is expected between neighboring ribosomes on an mRNA (19), the time gap between ribosomes will be roughly $\tau = 0.68s$ on average. Hence, when a ribosome encounters a stop codon, the total processing time to: (1) release the peptide, (2) recycle RF1/RF2 and RF3, and (3) split the ribosome by RRF and EF-G must take on average about $0.6 - 0.7s$ to avoid upstream ribosome queuing behind the terminating ribosome, resulting in poor translation efficiency. With the rates and model proposed from earlier work (29–31), the total processing time would be on the order of $5 - 7$ s, that is far too long to handle the expected density of ribosome loading on mRNAs at high bacterial growth rates. Fixing the model would primarily require unrealistic increases in RF3 concentration (5-10 fold) and a large increase in RF3 GTP hydrolysis rates ($>$ 10 fold).

Recent measurments on class 1 and class 2 release factors and their order of release have presented a possible solution to this timing problem. The latest experimental measurments on class 1 and class 2 release factors have suggested that the order of release of RF3 and RF1/RF2 may be random, as the off rates are similar (33, 37, 39). Thus, GTP hydrolysis by RF3 may only be required to recycle RF3 if RF1/RF2 have dissociated before RF3, resulting in a stalled complex (33, 39). Moreover, Adio *et al.* have also shown that the rate of dissociation of RF2 from the postHC appears independent of the presence of RF3, while for RF1, RF3 accelerates dissociation of RF1 by at least 10 fold (33). These observations, coupled with the fact that RF3 is a non-essential protein in many bacteria (20, 21), suggests that the kinetic model of RF1/RF2 recycling should include an RF3-independent pathway as well as a pathway in which RF3:GTP can leave prior to RF1/RF2 without requiring GTP hydrolysis. I have incorporated these features into a kinetic model of RF1/RF2 recycling which follows the model of Adio *et al.* (33). The kinetic rates from Adio (33) result in much better timings of RF1/RF2 recycling (around 1 s). However, some minor

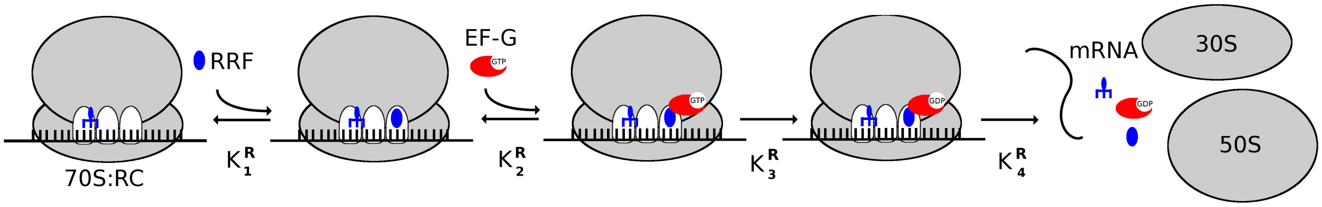

Figure G. Kinetic model of 70S splitting. The kinetic reactions during by ribosomal splitting by ribosomal recycling factor RRF and Ef-G are shown. Kinetic rates are listed in Table I.

adjustments to the rates must still be made in order for the overall estimated recycling time for a ribosome to be consistent with the minimal spacing between ribosomes, but these adjustments are much smaller then would be required in the Zavialov model (31).

To adjust the rates, I have started with kinetic rates for ribosomal ratcheting of $3-6s^{-1}$, release of RF1/RF2 + RF3 of $1.3s^{-1}$, and GTP hydrolysis of $1s^{-1}$, which are consistent with the ranges reported by Adio and Koutmou *et al.* (33, 37). As noted above, these parameters give recycling times on the order of 1-2 s, considerably better then the Zavialov model, but less than the time needed to prevent stalling of upstream ribosomes. Using these values as starting kinetic rates, I have adjusted each of them by a factor e^{-dx_i} given the constraint that $\sum_i (dx_i)^2$ is minimized and the total mean first passage time of RF1/RF2 recycling is 0.30 s, similar to the procedure done in Rudorf for the elongation rates (42). Given that Borg *et al.* (22) have estimated an *in vivo* RRF Ef-G mediated splitting time of $0.2s$, this brings the total processing time for ribosomal recycling (i.e. peptide release $\approx$ 0.1s, RF1/RF2 recycling $\approx$ 0.3s, and RRF mediated spliting$\approx$ 0.2s) to $\approx 0.60s$. This is well within the processing rate required for the highest expected ribosomal densities on mRNA (19).

Figure F details the kinetic model of RF1/RF2 recycling I have used in my model, while Figure G gives the kinetic model I have used for 70S splitting by RRF and EF-G. The latter closely follows the kinetic scheme of Borg *et al.* (22) which has an estimated splitting time *in vivo* of 0.2 s. Kinetic parameters used for both models are given in Tables H and I, respectively.

Model of GDP/GTP Exchange on GTPases. During ribosome initiation, elongation, and recycling, there are a number of kinetic steps which perform GTP hydrolysis and consume GTP as a result. In particular, IF2 during initiation, Ef-Tu and Ef-G during elongation, and RF3 during termination and recycling. After GTP hydrolysis, GDP will remain bound to these protein GTPases and GDP must be exchanged for GTP in order for these factors to be able to perform these elongation, initiation, and recycling steps again. The GTPases Ef-G and IF2 are not believed to have a guanine nucleotide exchange factor, and thus GDP is exchanged for GTP via the normal on/off kinetics for the guanine nucleotides. However, it should be noted that Zavialov *et al.* suggested that the ribosome could act as a guanine exchange factor for Ef-G (23). This was due to their estimates of the dissociation constants for Ef-G GDP and Ef-G GTP, suggesting that Ef-G would be predomantely in the GDP-bound state in the cell. Subsequent measurements of the dissociation constant for Ef-G GTP by Wilden *et al.* (44) showed that the dissociation constant for Ef-G GTP was much lower than previously measured, and that the ribosome does not accelerate GDP unbinding from Ef-G. Therefore, they proposed that the ribosome does not act as a guanine exhange factor for Ef-G. My model confirms that GDP can be exchanged for GTP sufficently quickly using the Wilden kinetic rates without the need for an additional guanine exchange factor. Table J lists the rates for Ef-G binding to GTP and GDP from Wilden *et al.* (44), and for IF2 binding to GTP and GDP from Mitkevich *et al.* (49).

Unlike Ef-G and IF2, there is a requirement for the guanine exchange factor Ef-Ts to mediate the exchange of GDP for GTP on Ef-Tu due to the long time that either GTP or GDP will be in complex with Ef-Tu before dissociation (i.e. 50-300 sec). I have used the models from Wieden and Schummer (25, 26) to create a GTP/GDP exchange reaction pathway in my model. A diagram of the kinetic reaction network is shown in Figure H, while Table J lists the rates.

Finally for RF3, there is some controversy about if the ribosome must act as a guanine exchange factor for the protein. Originally, Zavialov (31) had measured off rate for GDP unbinding from RF3 to be about $0.032s^{-1}$ with a dissociation constant of about $5nM$. Their measurement for GTP unbinding found a much higher dissociation constant of about $2.5\mu M$, suggesting RF3 would be in the GDP-bound form in the cell. Zavialov additionally found that the release of GDP was accelerated when RF3:GDP was bound to a postHC ribosome containing RF1/RF2. This led to the hypothesis that the ribosome was acting as a gunanine exchange factor for RF3, similar to their previous hypothesis for Ef-G (23). Subsequently, Peske *et al.* measured dissociation of GTP and GDP from RF3 and the effect of the ribosome on GDP dissocation (38). They found similar values for the dissociation constant for RF3:GDP of $K_d = 5nM$, but found a much lower dissociation constant for RF3:GTP of $K_d = 20nM$, and gave off rates in the range of $0.15s^{-1}$ for GDP unbinding from free RF3. From these measurments, they concluded that the majority of RF3 would be GTP-bound in the cell (38). Further measurements by Koutmou *et al.* (37) confirmed these dissociation constants and off rates. Interestingly, they found that, as with Zavialov (31), that GDP dissociation was accelerated (roughly to $35s^{-1}$) when RF3:GDP was bound to a postHC ribosome containing RF1/RF2. However, due to GTP being present in the cell at roughly 7-10 times GDP, they concluded that while GDP exchange for GTP on the ribosome was possible, it was a rare pathway due to RF3 being predominately in the GTP bound form. I have found that, without implementation of a kinetic pathway that allows GDP exchange for GTP on RF3 when it is bound to the ribosome, the

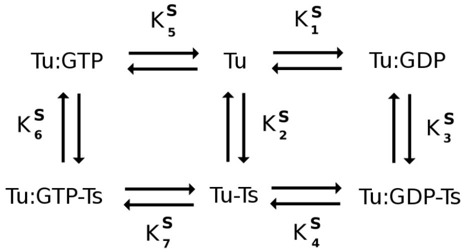

Supporting Figure H. Kinetic model for GTP/GDP binding and exchange on Ef-Tu. The kinetic reactions for Ef-Ts mediated exchange of GDP for GTP are illustrated. Kinetic rates are listed in Table J.

RF3:GDP form increases in concentration untill it is the vast majority of RF3 guanine nucleotide complexes. This is due to the slow rate of spontaneous GDP dissociation from free RF3:GDP, which takes on average 6 seconds to occur (assuming a dissociation rate of $0.15s^{-1}$) and the number of recycling events that occur. The slow spontaneous dissociation can not cope with the production of RF3:GDP coming from the RF3 GTP hydrolysis pathway (c.f. kinetic rates K_6^T and K_7^T in Figure G). Thus, I have added this GDP/GTP exchange pathway to the model and have found that removal of this exchange pathway results in ribosomal stalling and slow recycling in simulations.

Estimation of *in vivo* Concentrations of Cellular Proteins

During protein translation of an mRNA by the ribosome, several cellular proteins are required to complete the full initiation, elongation, and recycling process. These are: (1) the initiation factors IF1-IF3, (2) the elongation factors Ef-Tu, Ef-Ts, and Ef-G, (3) the three release factors RF1-RF3 and recycling factor RRF. In addition to concentrations of these proteins, estimates of the concentrations of up to 64 tRNAs along with the concentrations of at least 20 aminoacyl-tRNA synthases which covalently link an amino acid to a specific tRNA, are needed to describe the consumption of amino-acids and recharging of tRNAs. Finally, since all of these processes consume ATP and GTP, accurate estimates of both the production rate of GTP and ATP and of the 20 amino-acids would also be required. To complicate matters, the concentrations of all of these proteins, amino-acids, tRNAs, and nucleotide tri-phosphates are not strictly constant, but are changing due to changes in the bacterial growth rate and the availability of resources from the environment. To circumvent this problem, I have assumed the scenario of quasi-steady state protein synthesis. Specifically, I make the assumption that the bacterial cell has sufficient resources available to achieve a growth rate of μ doublings every 60 minutes, and that the *total* quantities of the main metabolites (ATP,GTP, amino-acids, and aminoacylated tRNAs) remain constant, along with the *total* numbers of cellular proteins. However, the free concentrations are allowed to change according to the set of kinetic reactions in the model.

I rely on a number of experimental studies which have been performed on *E. coli* at different growth rates to estimate the *total* quantities of various metabolites and cellular proteins. While these experiments present an estimate of the amounts of different cellular proteins that can be present at different growth rates, it should be noted that these are only rough estimates as these experiments were done at different growth conditions, (LB media vs TB media or glucose rich media etc.), which likely has a complex effect on the concentration of these factors. However, for the quantites used (see Tables K-N), I have been able to reproduce observed translational rates and behaviours of the ribosome that are expected *in vivo* (19).

Initiation, Elongation, and Release Factors. To determine the amount of total numbers of initiation factors (IF1,IF2,and IF3), elongation factors (Ef-Tu, Ef-Ts, and Ef-G), and release factors (RF1,RF2,RF3,and RRF) that are present in a bacterial cell, I have used estimates from Bremer (19), combined with recent liquid chromotography-mass spectrometry experiments performed by Schmidt *et al.* on the proteome of *E. coli* (27). Bremer give estimates of the numbers of IF1, IF2 and IF3, as well as elongation factors, in terms the number of ribosomes at a doubling time of $\tau = 40$ min, while Schmidt *et al.* give estimates of the total protein copy numbers per cell based on different growth rates of *E. coli* in a chemostat. It has been noted that elongation factors and initiation factors have an approximately linear scaling with the total number of ribosomes in the cell (17, 18), and I follow this scaling here for different growth rates. I have relied on measurements of Schimdt reported at $\mu = 0.5$ to estimate the numbers of IF1-IF3, EF-G, EF-Tu and RF1-RF3 per ribosome. Using 30,000 ribsomes, their values of the various factors per ribosome are in close agreement with estimates from Bremer and Dennis (19). Table K gives the individual numbers of initiation factors in an *E. Coli* cell for different growth rates derived by using the number per ribosome factors of Schmidt (27). I have found that these concentrations work quite reliabily in the model. However, there is one exception. The amount of Ef-Ts (0.35 per ribosome in Schmidt and 0.18 per ribosome in Bremer (19)) is too low to effectively cycle the Ef-Tu:GDP being produced during translation back to Ef-Tu:GTP. I have determined the optimal value, which maximizes free ternary complex in the $\mu = 2.5$ simulation, to be around 0.6 per ribosome. Lower concentrations of Ef-Ts fail to effectively exchange GDP for GTP at the rate it is being produced, while higher concentrations tends to shift more Ef-Tu to be in complex with Ef-Ts. Finally, it should be noted that it may be that the concentrations of RF1 and RF2 reported by Schmidt may be too low as well. This is due to the rate of premature termination on the ribosomes being more infrequent than expected. More experimental work on the rate of premature termination and ribosomal readthrough on stop codons is needed to better determine the concentrations of these proteins and rates of termination.

tRNAs. As discussed above in the model for the kinetic reactions of the 70S elongation complex, codon/anti-codon recognition in *E. coli* occurs via 41 tRNAs which may have cognate recognition with multiple codons (16), and these cognate interactions have been incoroporated into the model via a 64×64 cognate/near-cognate recognition matrix. To determine quantites of the individual 41 tRNAs in the cell at different growth rates, I have used the molar estimates from Dong *et al.* (16) and normalized these to the total tRNA concentration in the cell given by Bremer (19). Individual numbers of tRNAs at different growth rates are given in Table L. Assuming 8k-60k ribosomes in the cell as reported in Table K, the quantity of tRNAs roughly scales linearly with the number of ribosomes, and the best fit scaling factor per ribosome for each tRNA is given in the last column. As discussed in the main text, efficient protein translation is dependant on the overall codon composition of the transcription coupled with the concentrations of the individual tRNAs. Thus, Table L should be taken as a rough estimate of overall tRNA abundance, which has been optimized for an unknown state of the transcriptome. The individual tRNA numbers for each growth rate in Table L was calcualted by taking the intracellular concntrations from Table 5 in Dong et al (16) and assuming a volume of an *E. coli* cell of $1um^3$. The overall tRNA abundance scales as roughly 9.0 per ribosome, which fits fairly well with the data reported in Table 3 in Bremer and Dennis (19).

Nucleotide Di,Tri-phosphates and Amino Acids. For the amounts of various nucleotide phosphates (mono,di,and tri-) as well as approximate concentrations of amino acids, I have used experimental studies from Bennett *et al.* that look at intracellular concentrations of various metabolites in glucose fed *E. coli* (48). The values used in the model are listed in Table M for amino acids, and Table N for ATP,GTP etc. As discussed previously, I assume that the total number of these metabolites remains constant during protein synthesis. Since GTP is being consumed and replaced by GDP during the elongation phase of the ribosome, I remove one GDP molecule and replace it with a GTP molecule after each GTP hydrolysis event. An alternative formulation of this procedure would be to add an additional GTP->GDP reaction using a Lagrangian constraint. To model the Lagrangian constraint properly, or alternatively model the metabolic pathway in more detail, this would require a full understanding of the metabolic pathways involved in GTP and ATP regeneration in the cell, which is beyond the scope of this work.

1. Milon P, Maracci C, Filonava L, Gualerzi CO, Rodnina MV (2012) Real-time assembly landscape of bacterial 30S translation initiation complex. *Nat Struc Mol Biol* 19(6):609-615.
2. de Smit MH, van Duin J (2003) Translational standby sites: how ribosomes may deal with the rapid folding kinetics of mRNA. *J Mol Biol* 331:737-743.
3. Salis HM, Mirsky EA, Voigt CA (2009) Automated design of synthetic ribosome binding sites to control protein expression. *Nat Biotech* 27(10):946-950.
4. Milon P, Rodnina MV (2012) Kinetic control of translation initiation in bacteria. *Critical Rev Biochem Mol Biol* 47(4):334-348.
5. Studer SM, Joseph S (2006) Unfolding the mRNA Secondary structure by the bacterial translation initiation complex. *Mol Cell* 22:105-115.
6. Grigoriadou C, Marzi S, Kirillov S, Gualerzi CO, Cooperman BS (2007) A quantitative kinetic scheme for 70S translation initiation complex formation. *J Mol Biol* 373:562-572.
7. Milon P, Konevega AL, Gualerzi CO, Rodnina MV (2008) Kinetic checkpoint at a late step in translation initiation. *Mol Cell* 30:712-720.
8. Tomsic J, Vitali LA, Daviter T, Savelsbergh A, Spurio R, Striebeck P, Wintermeyer W, Rodnina MV, Gualerzi CO (2000) Late events of translation initiation in bacteria: a kinetic analysis. *EMBO Journal* 19(9):2127-2136.
9. Goyal A, Belardinelli R, Maracci C, Milon P, Rodnina MV (2015) Directional transition from initiation to elongation in bacterial translation. *Nucl Acids Res* 43(22):10700-10712.
10. Rodnina MV (2018) Translation in Prokaryotes. *Cold Spring Harb Perspect Biol* doi:10.1101/cshperspect.a032664.
11. Hecht A, Glasgow J, Jaschke PR, Bawazer LA, Munson MS, Cochran JR, Endy D, Salit M (2017) Measurements of translation initiation from all 64 codons in *E. coli*. *Nuc Acids Res* 45(7):3615-3626.
12. Borg A, Ehrenberg M (2015) Determinants of the rate of mRNA translocation in bacterial protein synthesis. *J Mol Biol* 427:1835-1847.
13. Ramakrishnan V (2002) Ribosome structure and the mechanism of translation. *Cell* 108:557-572.
14. Mathews DH, Sabina J, Zuker M, Turner DH (1999) Expanded sequence dependence of thermodynamic parameters improves prediction of RNA secondary structure *J Mol Biol* 288(5):911-940.
15. Calander R (2006) The bacteriophages. Second Edition. Oxford University Press
16. Dong H, Nilsson L, Kurland CG (1996) Co-variation of tRNA Abundance and Codon Usage in *Escherichia coli* at Different Growth Rates. *J Mol Biol* 260:649-663.
17. Pedersen S, Bloch PL, Reeh S, Neidhardt FC (1978) Patterns of protein synthesis in E. coli: a catalog of the amount of 140 individual proteins at different growth rates. *Cell* 4(1):179-90
18. Howe J, Hershey J (1983) Initiation factor and ribosome levels are coordinately controlled in *Escherichia coli* growing at different rates. *J Biol Chem* 258:1954–1959.
19. Bremer H, Dennis PP (2008) Modulation of Chemical Composition and Other Parameters of the Cell at Different Exponential Growth Rates. *Ecosal Plus 2013*; doi: 10.1128/ecosal.5.2.3
20. Zaher HS, Green R (2011) A primary role for release factor 3 in quality control during translation elongation in Escherichia coli. *Cell* 147(2):396-408.
21. Pavlov MY, Freistroffer DV, MacDougall J, Buckingham RH, Ehrenberg M (1997) Fast recycling of *Escherichia coli* ribosomes requires both ribosome recycling factor (RRF) and release factor RF3. *EMBO Journal* 16(13):4134-4141.
22. Borg A, Pavlov M, Ehrenberg M (2017) Complete kinetic mechanism for recycling of the bacterial ribosome. *RNA* 22:10-21.
23. Zavialov AV, Hauryliuk VV, Ehrenberg M (2005) Guanine-nucleotide exchange on ribosome-bound elongation factor G initiates the translocation of tRNAs. *J Biology* 4:9.
24. Andreeva I, Belardinelli R, Rodnina MV (2018) Translation initiation in bacterial polysomes through ribosome loading on a standby site on a highly translated mRNA. *Proc Nat Acad Sciences* 115(7):4411-4416.
25. Wieden H-J, Gromadski KB, Rodnin D, Rodnina MV (2002) Mechanism of EF-Ts-Catalyzed guanine nucleotide exchange in EF-Tu: Contribution of interactions mediated by helix B of EF-Tu. *Journal of Biol Chem.* 277(8):6032-6036.
26. Schummer T, Gromadski KB, Rodnina MV (2007) Mechanism of EF-Ts-Catalyzed guanine nucleotide exchange in EF-Tu: Contribution of interactions mediated by helix B of EF-Tu. *Biochemistry* 46:4977-4984.
27. Schmidt A, et al. (2015) The quantitative and condition-dependent Escherichia coli proteome. *Nature Bio* 34(104):3418.
28. Freistroffer DV, Kwiatkowski M, Buckingham RH, Ehrenberg M (2000) The accuracy of codon recognition by polypeptide release factors. *Proc Nat Acad Sci* 97(5):2046-2051.
29. Freistroffer DV, Pavlov MY, MacDougall J, Buckingham RH, Ehrenberg M (1997) Release factor RF3 in *E. coli* accelerates the dissociation of release factors RF1 and RF2 from the ribosome in a GTp-dependant manner. *EMBO* 16(13): 3126-4133.
30. Zavialov AV, Mora M, Buckingham RH, Ehrenberg M (2002) Release of peptide promoted by the GGQ motif of class 1 release factors regulates the GTPase activity of RF3. *Mol Cell* 10:789-798.
31. Zavialov AV, Buckingham RH, Ehernberg M (2001) A posttermination ribosomal complex is the guanine nucleotide exchange factor for peptide release factor RF3. *Cell* 107:115-124.
32. Hetrick B, Lee K, Simpson J (2009) Kinetics of stop codon recognition by release factor 1. *Biochem* 48:11178-11184.
33. Adio S, Sharma H, Senyushkina T, Karki P, Maracci C, Wohlgemuth I, Holtkamp W, Peske F, Rodnina MV(2018) Dynamics of ribosomes and release factors during translation termination in *E. coli eLife* 7:e34252.
34. Gao H, Zhou Z, Rawat U, Huang C, Bouakaz L, Wang C, Cheng Z, Liu Y, Zavialov A, Gursky R, Sanyal S, Ehrenberg M, Frank J, Song H (2007) RF3 induces ribosomal conformational changes responsible for dissociation of class 1 release factors. *Cell* 129:929-941.
35. Kuhlenkoetter S, Wintermeyer W, Rodnina MV (2011) Different substrate-dependant transition states in the active site of the ribosome. *Nature* 476:351-355.
36. Indrisiunaite G, Pavlov MY, Heurgue-Hamard V, Ehrenberg M (2015) On the pH dependance of class-1 RF-dependant termination of mRNA translation. *J Mol Biol* 427:1848-1860.
37. Koutmou KS, McDonald ME, Brunelle JL, Green R (2014) RF2:GTP promotes rapid dissociation of the class 1 termination factor. *RNA* 20:609-620.
38. Peske F, Kuhlenhoetter S, Rodnina MV, Wintermeyer W (2014) Timing and hydrolysis by translation termination factor RF3. *Nucl Acids Res* 42(3):1812-1820.
39. Shi X, Simpson J (2016) Mechanism of translation termination: RF1 dissociation follows dissociation of RF3 from the ribosome. *Biochem* 55:6344-6354.
40. Pape T, Wintermeyer W, Rodnina MV (1998) Complete kinetic mechanism of elongation factor Tu-dependant binding of aminoacyl-tRNA to the A site of the *E. coli* ribosome. *EMBO* 17(24):7490-7497.
41. Rodnina MV, Fischer N, Maracci C, Stark H (2017) Ribosome dynamics during decoding. *Phil Trans R Soc B* 372:20160182.
42. Rudorf S, Thommen M, Rodnina MV, Lipowsky R (2014) Deducing the kinetics of protein synthesis *in vivo* from the transition rates measured *in vitro*. *PLOS Comp Biol* 10(10):e1003909.
43. Rodnina MV, Savelsberg A, Katunin VI, Wintermeyer W (1997) Hydrolysis of GTP by elongation factor G drives tRNA movement on the ribosome. *Nature* 385:37-41.

44. Wilden B, Savelsberg A, Rodnina MV, Wintermeyer W (2006) Role and timing of GTP binding and hydrolysis during EF-G-dependant tRNA translocation on the ribosome. *Proc Nat Acad Sci* 103(37):13670-13675.
45. Katunin VI, Savelsberg A, Rodnina MV, Wintermeyer W (2002) Coupling of GTP hydrolysis by elongation factor G to translocation and factor recycling on the ribosome. *Biochem* 41:12806-12812.
46. Savelsberg A, Katunin VI, Mohr D, Peske F, Rodnina MV, Wintermeyer W (2003) An elongation factor G-induced ribosome rearrangement precedes tRNA-mRNA translocation. *Mol Cell* 11:1517-1523.
47. Morgan S, Higgs P (1998) Barrier heights between ground states in a model of RNA secondary structure. *J Phys A: Math Gen* 31:3153-3170.
48. Bennett BD, Kimball EH, Gao M, Osterhout R, Van Dien SJ, Rabinowitz JD (2009) Absolute Metabolite Concentrations and Implied Enzyme Active Site Occupancy in *Escherichia coli*. *Nat Chem Biol* 5(8):593.
49. Mitkevich VA, Shyp V, Petrushanko IY, Soosaar A, Atkinson GC, Tenson T, Markarov AA, Hauryliuk V (2012) GTPases IF2 and EF-G bind GDP and the SRL RNA in a mutually exclusive manner. *Sci Reports* 2:843.
50. Sin C, Chiarugi D, Valleriani A (2016) Quantitative assessment of ribosome drop-off in *E. coli*. *Nuc Acids Res* 44(6):2528-37.

Table A. Kinetic rates for 30S:PIC formation. Rates for binding of the initiation factors and fMet-tRNA to the ribosomal 30S subunit during formation of the 30S:PIC (c.f. Figure A) have been exerimentally measured from FRET fluorescence assays in (1).

Reaction	Label	On Rate $(1/\mu Ms)$	Off Rate $(1/s)$	Ref.
IF3 Binding	K_1^P	1160	36.0	(1)
IF2:GTP Binding	K_2^P	280	12.0	(1)
IF1 Binding	K_3^P	12	0.02	(1)
fMet Binding	K_4^P	5	1.50	(1)

Table B. Kinetic rates for 30S:PIC binding to mRNAs and joining of the 50S. Formation of the 30S:IC have been exerimentally measured from FRET fluorescence assays (5) for binding to both unstructured and structured mRNAs, while rates for 50S binding to the 30S:IC have been exerimentally measured from fluorescence assays by Grigoriadou (6).

Reaction	Label	On Rate $(1/\mu Ms)$	Off Rate $(1/s)$	Ref.
30S:PIC Standby mRNA Binding	K_1^B	25	25.0	(5)
30S:PIC Unstruc mRNA Binding	K_2^B	250	2.50	(5)
50S Binding to 30S:IC-GTP	K_1^I	34	35	(6)
50S Binding to 30S:IC-GDP	K_2^I	4.8	12	(6)

Table C. Kinetic rates for 70S Initiation. Rates have been exerimentally measured from fluorescence assays by Grigoriadou (6) and by Goyal (9).

Reaction	Label	Forward Rate $(1/s)$	Backward Rate $(1/s)$	Ref.
GTP Hydrolysis	K_3^I	44	–	(6)
Conf. Switch	K_4^I	24.0	2.10	(6)
Pi Release	K_5^I	3.7	–	(9)
IF1/IF2 Release	K_6^I	6.0	–	(9)
IF3 Release	K_7^I	3.2	–	(9)
70S Maturation	K_8^I	6.2	–	(9)

Table D. Kinetic rates for 70S Elongation - cognate tRNAs. Rates have been obtained from Rudorf *et al.* (42) and Borg *et al.* (22) for Ef-G binding. *Rates K_8^E - K_{10}^E are related to the single rate ω_{pro} in (42).

Reaction	Label	Forward Rate	Backward Rate	Ref.
aa-tRNA Binding	K_1^E	94	2300	(42)
Codon Recognition	K_2^E	3300	2	(42)
EFTu:GTP Hydrolysis	K_3^E	1700	—	(42)
EF-Tu Rearrangement	K_4^E	540	—	(42)
aa Accommodation	K_5^E	350	—	(42)
aa-tRNA Rejection	K_6^E	1	—	(42)
EF-G Binding	K_7^E	70	35	(22)
EFG:GTP Hydrolysis	K_8^E	250	—	*
Translocation	K_9^E	300	—	*
EF-G/tRNA Ejection	K_{10}^E	200	—	*

Table E. Kinetic rates for 70S Elongation - near-cognate tRNAs. Rates have been obtained from Rudorf *et al.* (42) and Borg *et al.* (22) for Ef-G binding. *Rates K_8^E - K_{10}^E are related to the single rate ω_{pro} in (42).

Reaction	Label	Forward Rate	Backward Rate	Ref.
aa-tRNA Binding	K_1^E	94	2300	(42)
Codon Recognition	K_2^E	3300	4300	(42)
EFTu:GTP Hydrolysis	K_3^E	4	—	(42)
EF-Tu Rearrangement	K_4^E	540	—	(42)
aa Accommodation	K_5^E	0.27	—	(42)
aa-tRNA Rejection	K_6^E	7	—	(42)
EF-G Binding	K_7^E	70	35	(22)
EFG:GTP Hydrolysis	K_8^E	250	—	*
Translocation	K_9^E	300	—	*
EF-G/tRNA Ejection	K_{10}^E	200	—	*

Table F. Kinetic rates for release factor 1 binding and hydrolysis. Release factor 1 binding and hydrolysis of the peptide bond have been measured by Freistroffer (28). For RF1 unbinding from UAA and UAG and subsequent hydrolysis of the peptide bond, rates are used from (33) and (36), respectively. Binding of RF1 to the sense codons AAA, AAG, GAA, and GAG was found to have very high off-rates making observed peptide release difficult to measure. Default values of 500 and 0.001 are used in these cases.

Codon	K_1^T $(\mu M^{-1}s^{-1})$	K_{-1}^T (s^{-1})	K_2^T (s^{-1})	Ref.
UAA	60.0	0.10	23.0	(28, 33, 36)
UAG	60.0	0.10	23.0	(28, 33, 36)
AAA	18.0	500.00	0.001	(28)
AAG	18.0	500.00	0.001	(28)
CAA	18.0	298.00	0.002	(28)
CAG	18.0	448.00	0.003	(28)
GAA	18.0	500.00	0.001	(28)
GAG	18.0	500.00	0.001	(28)
UAC	18.0	176.00	0.029	(28)
UAU	18.0	60.00	0.200	(28)
UCA	18.0	149.00	0.034	(28)
UCG	18.0	148.00	0.026	(28)
UGA	18.0	358.00	0.009	(28)
UGG	18.0	186.00	0.013	(28)
UUA	18.0	179.00	0.022	(28)
UUG	18.0	149.00	0.041	(28)

Table G. Kinetic rates for release factor 2 binding and hydrolysis. Release factor 2 binding and hydrolysis of the peptide bond have been measured by Freistroffer (28). For RF2 unbinding from UAA and UGA and subsequent hydrolysis of the peptide bond, rates are used from (33) and (36), respectively. Binding of RF2 to the sense codon AAA was found to have very high off-rates making observed peptide release difficult to measure. Default values of 500 and 0.001 are used in this case.

Codon	K_1^T $(\mu M^{-1}s^{-1})$	K_{-1}^T (s^{-1})	K_2^T (s^{-1})	Ref.
UAA	23.0	1.300	23.0	(28, 33, 36)
UGA	23.0	1.300	23.0	(28, 33, 36)
AAA	18.0	500.00	0.001	(28)
AGA	18.0	344.00	0.001	(28)
CAA	18.0	522.00	0.016	(28)
CGA	18.0	285.00	0.024	(28)
GAA	18.0	308.00	0.001	(28)
GGA	18.0	344.00	0.003	(28)
UAC	18.0	594.00	0.050	(28)
UAG	18.0	295.00	0.210	(28)
UAU	18.0	594.00	0.018	(28)
UCA	18.0	2495.0	0.480	(28)
UGC	18.0	594.00	0.044	(28)
UGG	18.0	368.00	0.190	(28)
UGU	18.0	507.00	0.029	(28)
UUA	18.0	142.00	0.008	(28)

Table H. Kinetic rates for RF1/RF2 recycling on the 70S:postHC. Rates have been measured in (33, 37) and have been modified to obtain an average mean first passage time for RF1/RF2 recycling of $0.3s$.

Reaction	Label	Forward Rate	Backward Rate
RF3 Binding	K_3^T	36.0	1.30
Ribosome Ratcheting	K_4^T	18.1	2.27
RF1 Release (+RF3)	K_5^T	13.4	–
RF2 Release (+RF3)	K_5^T	13.4	–
GTP Hydrolysis	K_6^T	5.0	–
RF3:GDP Release	K_7^T	15.5	–
RF3:GTP Release	K_8^T	13.4	–
RF1 Release (-RF3)	K_9^T	0.2	–
RF2 Release (-RF3)	K_9^T	2.0	–

Table I. Kinetic rates for ribosome splitting. Rates for postHC ribosomal splitting by RRF have been measured in Borg *et al.* (22).

Reaction	Label	Forward Rate	Backward Rate	Ref.
RRF Binding	K_1^R	15.5	58.0	(22)
EF-G Binding	K_2^R	70.0	35.0	(22)
GTP Hydrolysis	K_3^R	43.0	–	(22)
Ribosome Splitting	K_4^R	25.0	–	(22)

Table J. Kinetic rates for GTP and GDP binding to various GTPases. The rates for GTP and GDP binding to the GTPases IF2, RF3, Ef-G and Ef-Tu have been taken from (49) for IF2, (44) for Ef-G, (37, 38) for RF3, and (26) for Ef-Tu.

Reaction	Label	On Rate $(1/\mu M s)$	Off Rate $(1/s)$	Ref.
IF2 [GDP Binding]	K_1^G	10.0	16.0	(49)
IF2 [GTP Binding]	K_2^G	10.0	67.0	(49)
EFG [GDP Binding]	K_3^G	7.50	300.0	(44)
EFG [GTP Binding]	K_4^G	0.58	13.0	(44)
RF3 [GDP Binding]	K_5^G	30.0	0.15	(38)
RF3 [GTP Binding]	K_6^G	10.0	0.20	(38)
RF3 [GDP Bind postHC]	K_7^G	30.0	35.0	(38)
Tu [GDP Binding]	K_1^S	2.0	0.002	(26)
Tu [GTP Binding]	K_5^S	0.5	0.030	(26)
Tu-Ts [GDP Binding]	K_4^S	14.0	125.0	(26)
Tu-Ts [GTP Binding]	K_7^S	6.0	85.0	(26)
Tu:GDP [Ts Binding]	K_3^S	60.0	350.0	(26)
Tu:GTP [Ts Binding]	K_6^S	30.0	60.0	(26)
Tu [Ts Binding]	K_2^S	10.0	0.030	(26)
TC Formation	K_{tc}	1.5	0.0013	(26)

Table K. Number of cellular proteins at different growth rates. The numbers of initiation factors, ribosomes, elongation factors, and recycling factors are given based on protemics data from (27). The numbers are determined for each protein by multiplying the ribosomal ratio in the last column with the total number of ribosomes at a given growth rate.

Cellular Factor	$\mu = 0.7$	$\mu = 1.06$	$\mu = 1.60$	$\mu = 2.5$	$1/R$
Ribosome	8000	15000	30000	60000	1.00
IF1	720	1350	2700	5400	0.09
IF2	1040	1950	3900	7800	0.13
IF3	1600	3000	6000	12000	0.20
EF-Tu	43200	81000	162000	324000	5.40
EF-Ts	4800	9000	18000	36000	0.60
EF-G	6400	12000	24000	48000	0.80
RF1	37	69	140	276	0.0046
RF2	370	690	1400	2760	0.046
RF3	160	300	600	1200	0.02
RRF	880	1650	3300	6600	0.11

Table L. Numbers of various tRNAs per cell and their codon recognition. Data for the number of tRNAs at different growth rates have been adapted from (16). These have been normalized to overall expected total tRNA concentrations discussed in Bremer (19) and fitted to an average number per ribosome.

tRNA	Codon Rec.	$\mu = 0.7$	$\mu = 1.06$	$\mu = 1.50$	$\mu = 2.5$	$1/R$
Lys	AAA,AAG	2340	4360	6760	16680	0.287
Asn	AAC,AAU	1320	2580	4720	11650	0.177
Thr4	ACA,ACU,ACG	1090	1930	3860	11020	0.149
Thr1+3	ACC,ACU	1420	2610	4200	9930	0.172
Thr2	ACG	680	1250	2060	4990	0.083
Arg4	AGA	800	1560	2520	5630	0.099
Ser3	AGC,AGU	1510	2690	4180	9060	0.173
Arg5	AGG	540	950	1900	3510	0.063
Ile2	AUA	2040	3930	7330	19780	0.282
Ile1	AUC,AUU	2040	3930	7330	19780	0.282
MetF	AUG	2440	4970	7520	22370	0.336
MetM	AUG	890	1720	3170	7080	0.114
Gln1	CAA	930	2150	2450	7000	0.125
His	CAC,CAU	750	1560	2590	7000	0.104
Gln2	CAG	1060	2060	3920	10020	0.145
Pro3	CCA,CCU,CCG	650	1310	1970	4090	0.078
Pro2	CCC,CCU	860	1340	3100	5990	0.098
Pro1	CCG	830	2080	2130	4270	0.104
Arg3	CGG	890	860	1750	3670	0.076
Arg2	CGU,CGC,CGA	5000	9230	18410	40890	0.640
Leu3	CUA,CUG	850	1550	2470	5070	0.098
Leu2	CUC,CUU	1190	2390	3650	9480	0.155
Leu1	CUG	5130	9950	16520	35500	0.632
Glu2	GAA,GAG	5360	9920	18690	46940	0.704
Asp1	GAC,GAU	2790	5000	9330	24720	0.364
Ala1B	GCU,GCA,GCG	4030	8350	13570	33540	0.539
Ala2	GCC	720	1380	2470	5710	0.092
Gly2	GGA,GGG	1230	2290	4200	8860	0.151
Gly3	GGC,GGU	5230	9940	15370	39920	0.660
Gly1a	GGG	1230	2290	4200	8860	0.151
Val1	GUA,GUG,GUU	4150	6570	14710	32610	0.500
Val2ab	GUC,GUU	1510	2980	4880	11530	0.193
Tyr1+2	UAC,UAU	2150	3830	7610	14760	0.256
Ser1	UCA,UCU,UCG	1910	3240	5400	11770	0.216
Ser5	UCC,UCU	890	1700	2850	6440	0.110
Ser2	UCG	350	690	1060	2310	0.042
Cys	UGC,UGU	1670	3100	5450	11300	0.201
Trp	UGG	950	1980	3210	8020	0.128
Leu5	UUA,UUG	1190	2410	2820	6040	0.136
Phe	UUC,UUU	1230	2540	3630	8170	0.153
Leu4	UUG	2170	4140	7480	14870	0.265

Table M. Intracellular concentrations of amino acids. Concentrations are given for all 20 amino acids for exponentially growing *E. coli*. (Adapted from Bennett (48)).

Amino Acid	Concentration (μM)
Leu	300
Phe	18
Lys	410
Arg	570
Gly	800
Val	4000
Glu	96000
Ile	300
Gln	3800
Thr	180
Trp	12
Asp	4200
Ser	68
Tyr	29
Asn	510
Ala	2600
Pro	390
Met	150
His	68
Cys	370

Table N. Intracellular concentrations of nucleotide phosphates. Concentrations are given for all four nucleotides for exponentially growing *E. coli*. (Adapted from Bennett (48)).

NxP	Concentration (μM)
ATP	9600
ADP	560
AMP	280
CTP	2700
CDP	—
CMP	360
GTP	4900
GDP	680
GMP	24
UTP	8300
UDP	180
UMP	—
