# Supplementary material for: A stochastic model for simulating ribosome kinetics in vivo
Source: PLoS Comput Biol. 2020 Feb 12;16(2):e1007618. doi: 10.1371/journal.pcbi.1007618 (PMC7015319; doi:10.1371/journal.pcbi.1007618)
Supplement: Attachment — Submitted filename: response.pdf [file pcbi.1007618.s002.pdf]

Dear Colleagues,

I would like to thank all the reviewers for their time and helpful comments. I have addressed each of their concerns and specific responses to each referee are given below. Changes made in the manuscript have been highlighted in red.

Reviewer #1: The manuscript develops a stochastic model of protein synthesis, which is used to simulate translation in E. coli cells. I have problems fully evaluating this manuscript, due in part to the fact that the important aspects of the work seem incompletely described, and that novelty is claimed in a way that I do not completely agree with.

1) The introduction gives the impression that previous work on modelling protein synthesis has relied almost exclusively on mathematical modelling via the TASEP, that very little work was done on stochastic modelling, and that none of the latter attempted genome-wide models of translation. This is factually untrue – a detailed Gillespie-based model of translation was published eg in 2007 (PMID 17897886) and this was used for modelling genome-wide translation in yeast (22965119, 23791185). The author even cites a more recent model as using a similar approach – simulating translation in the cell or in a cell-free extract into fundamentally different apart from the parameterisation. The principal findings in the publications cited above on translation in vivo do not seem fundamentally different from those described here.

RESPONSE:

I apologize for leaving out references to the above works, which was an error on my part, and resulted in the referee misunderstanding the level of complexity which is included in the model that I report here. This has given the impression that I was implying no other stochastic work has been done on ribosome kinetics. There are some important differences between the stochastic models that are highlighted by the referee and my model, which I summarize below. Hopefully this clarifies the differences between my model and other stochastic models. Factors discussed below, such as Ef-G and Ef-Tu etc., are with reference to the bacterial ribosome and there will be equivalent factors (eEF1 and eEF2) in the yeast system. I have added a discussion of other stochastic models in the manuscript and highlighted the differences with the one reported here. However, please find detailed comments to your questions in points a-d below.

(a) The movement of the ribosome on the mRNA, and the consumption of tRNAs, are not the only processes related to the ribosome that are occurring in the cell. In addition, there are many subprocess that need to occur in order for the ribosome to have sufficient substrate to synthesise protein. Two examples of these subprocesses are (1) the re-charging of tRNAs and Ef-Tu and reformation of ternary complex, and (2) exchange of GTP/GDP on GTPases such as Ef-G and other factors. Although the stochastic models that are highlighted by the referee above do simulate ribosome kinetics of multiple ribosome on multiple mRNAs, they do not include all of these subprocess and enforce or simplify some of the kinetics, such as the kinetic steps for the elongation reactions. In contrast, all of the kinetic steps of elongation, and its dependence on Ef-G concentration and ternary complex concentrations, along with recharging reactions, are considered in the model reported here. Although the model in (Chu et al. PMID 17897886) takes into account tRNA concentration, there is only a single recharging reaction for the tRNAs and re-charging of Ef-Tu and formation of ternary complex is not considered. The result of neglecting the Ef-Tu recharging and formation of ternary complex is that the relative concentrations of the tRNAs will be approximately constant over the simulations with a

small dependency on the codon bias of the transcriptome. In contrast, taking formation of ternary complex, and the re-charging of Ef-Tu by Ef-Ts into account, the relative concentrations of tRNA **in ternary complex** is dynamic and can shift over time as a result of the codon bias of the transcriptome (see Figure 4 in the manuscript). This is because as tRNAs are consumed, they must be recharged and then rebound to new Ef-Tu:GTP to form new ternary complex resulting in a competition between charged tRNAs for formation of ternary complex. Underused tRNAs will have an advantage over overused ones in formation of TC, unless consumption of tRNAs matches codon bias of the transcriptome. This can result in starvation for certain amino acids, even though there is an abundance of the amino acid and charged tRNAs in the cell. As a result, the overall effects on translation speed (aa incorporated per second) are much stronger than previously appreciated. This was one of the main results of the model and demonstrates how removal of certain reactions can have dramatic effect on the results.
The comment from the referee

The principal findings in the publications cited above on translation in vivo do not seem fundamentally different from those described here.

Is incorrect because the different and new result is the strong impact that Ef-Tu recharging reactions have on the translation process and its implications on the regulation of tRNA concentration in the cell. To demonstrate the implications of removing the Ef-Tu recharging reactions (and formation of TC) on the translational process, I have removed these reactions from my model and allowed consumed tRNAs to immediately reform into TC after release from the ribosome. This is equivalent to what was done by Vieira (2016 BioPhys J, 110, 2120) and similar to that of (PMID = 22965119, 23791185). I have re-done the calculations on T10a (which has codon bias following E coli proteome) and T10 (which has an artificial codon bias following the tRNA propensities of Dong.) Both simulations were run using tRNA abundances from Dong. The result is that, once the Ef-Tu recharging reactions are removed, translation on the T10a is now much more efficient (with average 17.7 aa/sec translation speed) and similar to T10 (with average 18.4 aa/sec). When Ef-Tu recharging reactions are included, the T10a has very poor translational efficiency (average 8.1 aa/sec translation speed over the transcriptome) compared with T10 (average of 18.1 aa/sec). Thus, when Ef-Tu recharging reactions are taken into account, tRNA abundances must match their usage in the transcriptome closely to avoid shifts in the relative concentrations of tRNA in TC. This is why I have used the artificial transcriptome T10 as there is not a precise measurement of tRNA abundances (as demonstrated by the mismatch between tRNA usage in Dong and codon bias in WT E. Coli). The easiest “fix” to demonstrate that mismatch between tRNA usage and codon bias was causing the slow translation when Ef-Tu recharging is accounted for was to alter the codon bias of T10 to match tRNA abundances in Dong.

(b) There is also a second observation which has not been reported before which relates to emergent behaviour of the translational system. It is well known experimentally that in bacteria, the translation rate (number of amino acids incorporated by the ribosome per second) increases from around 15 aa/sec at a growth rate of 0.7 doublings per hour to 22 aa/sec at a growth rate of 2.5 doublings per hour. This feature emerges from the model on its own accord. Specifically, it is not enforced at all in the parameters of the model and manifests simply from the complex interactions between all of the components in the system. To my knowledge, there is no comprehensive model of translation which predicts/observes this feature. Indeed, the study by Vieira (2016 BioPhys J, 110, 2120) showed that there was no increase, contrary to experiment. This was due to the fact that the left out the effects of Ef-Tu recharging and recycling factor effects. Once both of these

are accounted for in the model, as is done here, the increase in translation speed emerges from the model. Second, the levels of free ribosome are not enforced in my model. These equilibrate naturally based on the complex interactions of the system. I believe, if I am not mistaken, that this feature is set/fixed using the parameter RATIO_OF_FREE_RIBOSOMES in the model from Chu et al. In this work, there is no such parameter and the number of free ribosomes equilibrates at 15% free ribosomes, consistent with experiment.

(c) The method that I have developed for stochastic simulation performs the exact Gillespie algorithm but does so in a way that results in a O(Log_2(N)) cost of selecting and firing a reaction instead of O(N) in the standard Gillespie algorithm. This results in a faster algorithm which can handle larger numbers of ribosomes more efficiently as the process scales as Log(N) with the N = number of ribosomes (or mRNAs) depending on how you choose objects in the system (see response to point 2 for further details).

(d) The model reported here is nearly complete, as it incorporates all known kinetic steps that occur during initiation, elongation, and recycling, and also accounts for the re-charging reactions occurring in the background (GDP exchange for GTP on Ef-Tu mediated by Ef-Ts for example) as well as pre-mature termination and other effects. I say nearly complete because, while the parameterization of the rates for elongation and recycling have been tested/compared with experimental observations, testing of the initiation model, and the incorporation of the full details of the aminoacylation process still remain. In the later, kinetic rates were determined experimentally for only one or two aminoacyl-tRNA-synthetase. Thus, until more measurements on additional aa-tRNA-synthetases are made, the incorporation of the full set of kinetic interactions associated with this step may be premature. A few additional differences between the model of (Chu et al. PMID 17897886) and the one reported here are (1) number of free ribosomes is not fixed (2) formation of ternary complex and recharging of Elongation factors are accounted for, (3) tRNA binding and mis-reading is a multi-step process (4) pre-mature termination and read-through events are allowed. Figure 1 of the manuscript details all the reactions taken into account. Since each of the “background” process such as Ef-G recharging and Ef-Tu recharging by Ef-Ts is taken into account and is concentration dependant, the model reported here can probe the full interplay between all the components (i.e. it is a complex system where the alteration of one factor can have profound effects on a different part of the system) and the effects of over/under-expression of Ef-Tu or other factors can be probed by the model reported here. One advantage of the model here is that it can predict expected free amounts of elongation factors which were previously estimated. One estimation (that roughly 90% of Ef-Tu is in free TC) is not consistent with the observations here which show that the amount is much smaller (only ~60-70% in free TC) because the remaining amount in complex with Ef-Ts for GTP/GDP exchange, or free for association with charged tRNA is higher than anticipated according to the kinetics of the model here.

2) Although I see strong overlap with previous work, there is some novelty in this manuscript but this is not clearly described. First, the detailed model for translation initiation is novel as far as I know, although this is qualified in the text as “somewhat hypothetical”. If there is uncertainty over the structure or parameterisation of this part of the model, this should have been discussed in more detail.

RESPONSE:

With regards to the parameters (elongation rates, recycling rates and quantities of proteins and tRNAs) these have been tested in this manuscript and shown that the parameters listed in the supporting information reproduce experimentally observed phenomenon, (1)

the increasing amino acid incorporation rate which occurs are higher E. Coli growth rates and (2) the levels of free ribosomes. The uncertainty in the initiation model is that these parameters have not been specifically tested against protein production levels for various mRNAs. Saying that the model is hypothetical is not a reference to the kinetic steps, as this is the best model based on experimental observations. My next plans are to test the initiation model with experimental observations for lacZ expression based on its 5'UTR and also look at the model's prediction of expression levels for phage MS2 coat protein based on the ribosome binding site of the coat gene. The latter was extensively studied by Van Duin and colleagues and will provide substantial data to compare with. I have added text in the manuscript to clarify that the uncertainty is related to the parametrization and further comparison with experimental measurements of protein levels are needed to firm these rates up.

Second, the technical approach used for the modelling described on page 4 seems interesting, but is described without any validation that this produces the same results as the original approach described by Gillespie, or as the faster derivatisations of this approach like tau-leap etc. In my view a more efficient algorithm for stochastic modelling of chemical systems would be very useful, but this would need to be validated by comparing results with more established approaches. I am assuming that the approach used here is more efficient than existing stochastic modelling approaches, if not, it should be explained why a new approach was necessary. It should also be explained how spatial information (ribosome positioning on the mRNAs) was encoded, as this is the biggest problem in standard ODE-based approaches and the reason why papers such as the one cited in the text require such high numbers of equations even for short mRNAs.

RESPONSE:
The "standard" Gillespie model is to (1) sum all reaction propensities to get a total reaction flux = Phi, (2) re-sum over all reactions until the partial sum of reaction propensities is > r*Phi with r in [0,1], picking the reaction that this occurs at and (3) update the reaction list and species. Thus, the standard Gillespie procedure requires a sum over N reactions at each step of the simulation and the overall computational cost scales as O(N). Tau-leaping approximates standard Gillespie by performing multiple reactions at once using a Poisson distribution to select the change in each chemical species. This is equivalent to making a large step tau in the time domain and comes at the price of accuracy. The procedure that I report here is exactly equivalent to the standard Gillespie procedure and is not an approximate scheme like the tau-leaping method. It performs the sum over all reaction propensities (step 1 of the procedure) using a binary tree so that the computational cost is Log_2(N) instead of O(N), speeding up the computation overall. The method does not require validation as it is equivalent to the standard Gillespie procedure by construction. All that the Log(N) method does is calculate the sum of the reaction propensities in a different order, which is an allowed transformation that will preserve the stochastic behaviour [see e.g. Cao 2004 J. Chem. Phys 121:4059]. Such techniques and related transforms, like the next reaction method, have been used by others to increase the efficiency of reaction selection and speed up the overall computation while maintaining a trajectory which is statistically equivalent to the standard Gillespie procedure. For example, [Cao Y, Li H, Petzold LR. 2004 J. Chem. Phys. 121:4059–67] have done reaction selection by ordering the reaction propensities from highest to lowest. This is exactly equivalent to standard Gillespie because, although the order of the reaction propensities is from largest to smallest, the random number remains unbiased. Gillespie (2007 Ann Rev. Phys Chem 58, 35-55) gives a review of alternative schemes for the standard Gillespie method which are equivalent but more efficient computationally. The method I report here is equivalent to standard Gillespie since the order of the sum of the reaction propensities is changed, as

was done by Cao and others, but just in a different manner. The exact method is detailed in the supporting info of (Dykeman, 2015 NAR, 43:5708) and was referenced in the text (ref 17).

The location of the ribosome on the mRNA is encoded based on the location of its P site. The computational code treats mRNAs as objects as opposed to treating ribosomes as objects as was done in Chu et al. This was done for technical reasons so as to be able to find neighbouring ribosomes up and downstream easily.

3) The approach for constructing the transcriptome appears so nonsensical that I wonder whether I misunderstand something fundamental. The author appears to say that they constructed the main transcriptome used in the simulations from proteome data, in such a way that the codon usage of the transcriptome fits the assumed tRNA pool better than experimentally reported transcriptomes. If I understood this correctly, they would model an entirely unphysiological transcriptome?

RESPONSE:

The transcriptome is based on E. coli proteins, but the codon bias has been altered to match the tRNA pool. Some discussion about the history of development of my model can hopefully clarify why I had to do this. When I was testing the parameters of the model and looking at matching the observed translation rates (15 aa/sec at 0.7 doubling/hour and 22 aa/sec at 2.5 doublings per hour) using **exact** mRNAs from E. coli taken directly from the uniprot database, the translation was very inefficient and average translation speed over the transcriptome was < 9 aa/sec (see transcriptome T10A) when it should be ~ 18 aa/sec for 1.0 doublings per hour growth rate. This was despite using kinetic rates from Rudorf et al. which were adjusted to the codon biases of E. coli and the tRNA abundances from the Dong et al experiment (see Rudorf et al. PLOS: Comp Biol. 10:e1003909). After examination of the model results, it became clear that certain tRNAs were not being recycled into new ternary complex very efficiently because other charged tRNAs, which were more abundant due to low demand from the transcriptome, were taking any available Ef-Tu:GTP and forming TC. This resulted in a lack of certain charged tRNAs in TC (see T10a of Figure 4 in the manuscript) and the translational machinery became starved for these tRNAs. It was then clear that the difference between Dong's measurements and the codon bias of E. Coli was enough to substantially alter the translation efficiency and resulted in most ribosomes being stalled. So as a proof that this was indeed the problem, I changed the codon bias of the E. Coli mRNAs to match the tRNA pool and re-simulated. In this situation, the model worked, and translation matched Rudorf's rates of 15 aa/sec and 18 aa/sec for 0.7 and 1.0 doublings per hour, respectively. Alternatively, if one neglects the reactions involving the recycling of Ef-Tu by Ef-Ts and formation of new TC and just assumes tRNA becomes recharged in a single reaction (as in Chu et al. PMID 17897886) or instantaneously in Vieira (2016 BioPhys J, 110, 2120), this problem largely goes away because there is no competition amongst the tRNAs for the finite resource of Ef-Tu needed to form TC. But if one tries to build a model that incorporates these reactions, then codon bias and tRNA usage must match fairly closely, otherwise the translational system *as a whole* doesn't work.

The problem is that to make the entire translational system work with authentic E. Coli mRNAs/transcriptome, the tRNA concentrations need to either be more accurately measured or adjusted using theoretical/computational methods. Measurement of the precise tRNA pool may be complicated because it could possibly be dynamic or linked with the transcriptome. On the other hand, the computational/theoretical approach is not a straightforward procedure as there are 43 tRNAs requiring a search in 43-dimensional

space. I have been currently working on optimizing the tRNA pool via several strategies while trying to match experimental measurements on tRNA-Lys misreading rates. So in a nutshell, the codon bias of the transcriptomes T10, T25, and T07 are altered to match the tRNA measurements in Dong, but the simulation with the authentic codon bias from proteome date (T10a) results in much less efficient translation, unless Ef-Tu recharging reactions are removed and modelled as instantaneous (see additional added simulations in the manuscript for proof).

Reviewer #2: In his manuscript, Dr. Dykeman presents to my knowledge the first computational model of in-vivo protein synthesis that is built on a close-to complete set of reactions and captures the stochastic nature of this highly complex process. He uses this model to study fundamental aspects of translation, such as the effect of the system's tRNA and codon compositions on the availability of free ternary complexes.
I find this work highly interesting and believe that it significantly advances the field. In particular, it is the first computational model of protein synthesis for a broad range of bacterial growth rates that uses only a single set of kinetic parameters. A convincing validation of the approach is given by comparing the numerical results with experimental observations.

The manuscript is very well organized and the text is very well written, clear and easy to follow. An exceptional comprehensive, thorough and transparent discussion about the limits of the model, which covers many challenges that are common for this type of modeling (e.g. a consistent parameterization), is included.

There is only one minor issue I kindly ask the author to comment on:
In the abstract you mention that "several biological phenomena are predicted as emergent properties of this complex translational dynamics". Could you please be a bit more specific so that the readers know what to expect from your paper?

RESPONSE:
Thank you for the kind response. I have added a sentence in the abstract to give details about the emergent properties.

Reviewer #3: The paper of Dykeman deals with the simulation of in vivo translation kinetics in E. coli outlining that the cellular context is issential to understand experimental findings.

While the approach is basically highly attractive and deserves publication, the paper has some shortcomings that need to be eliminated before publication:

1) The author outlines the in vivo character of the simulation. However, the reader cannot understand how (and why) the arbitrary transcriptomes have been designed (lines 154 -156). There are numerous E. coli publications covering genome-scale transcript data. Why should the transcript artifact of the author be superior to experimental measurements?

RESPONSE:
Thank you for the kind response. Referee 1 had a similar comment and the background history of the development of the model will explain why I have performed simulations with an artificial transcriptome. When I was testing the parameters of the model and looking at matching the observed translation rates (15 aa/sec at 0.7 doubling/hour and 22 aa/sec at 2.5 doublings per hour) using **exact** mRNAs from E. coli taken directly from the uniprot database, the translation was very inefficient and average translation over the transcriptome was < 9 aa/sec (see transcriptome T10a) when it should be ~ 18 aa/sec for 1.0 doublings per hour growth rate. This was despite using kinetic rates from Rudorf et al. which were adjusted to the codon biases of E coli and the tRNA abundances from the Dong et al. experiment (see Rudorf et al. PLOS: Comp Biol. 10:e1003909). After examination of the model's results, it became clear that certain tRNAs were not being recycled into new ternary complex very efficiently because other tRNAs, which were more abundant due to low demand from the transcriptome, were taking any available Ef-Tu:GTP and forming TC. This resulted in a lack of certain tRNAs in TC (see T10a of Figure 4 in the manuscript) and the translational machinery became starved for these tRNAs. It was then clear that the difference between Dong's measurements and the codon bias of E. Coli was enough to substantially alter the translation efficiency and resulted in most ribosomes being stalled. To prove that this was the problem, I changed the codon bias of the E. Coli mRNAs to match the tRNA pool and re-simulated. In this situation, the model worked, and translation matched Rudorf's rates of 15 aa/sec and 18 aa/sec for 0.7 and 1.0 doublings per hour, respectively. Alternatively, if one neglects the reactions involving the recycling of Ef-Tu by Ef-Ts and formation of new TC and just assumes tRNA becomes recharged in a single reaction (as in Chu et al. PMID 17897886) or instantaneously in Vieira (2016 BioPhys J, 110, 2120), this problem largely goes away because there is no competition amongst the tRNAs for the finite resource of Ef-Tu needed to form TC. But if one tries to build a model that incorporates these reactions, then codon bias and tRNA usage must match fairly closely, otherwise the translational system *as a whole* is inefficient.

Thus, it is not that the transcriptomes that I use in the paper are superior in anyway per se, it is just that in order to get the simulation to work with an experimental measured transcriptome, I would need to adjust tRNA concentrations to match the demand required by the transcriptome. The problem is that in practice, a naive matching of tRNA to codon bias does not work, most likely I believe because of the cross-recognition of some codons (e.g. tRNA-Pro3 recognizes CUA and CUG and CUU, but tRNA-Pro2 also recognizes CUC and CUU) providing a complex redundancy which needs accounting for. To make the entire translational system work with authentic E. Coli mRNAs/transcriptome, the tRNA concentrations need to either be more accurately measured or adjusted using theoretical/computational methods. Measurement of the precise tRNA pool may be

complicated because it could possibly be dynamic or linked with the transcriptome. On the other hand, the computational/theoretical approach is not a straightforward procedure as there are 43 tRNAs requiring a search in potentially 43-dimensional space. I have currently been working on optimizing the tRNA pool via several optimization strategies so that the model can be applied to authentic E. Coli mRNAs.

2) The adaptation of nucleotide sequence - apparently a crucial step for sequenced based modelling - is not properly explained (lines 158 - 159). Actually, the need to do so does not get clear at all.

RESPONSE:
Thank you for pointing out this unclear area of the text. Hopefully the answer to your prior question has answered most of how this was done. However, I have changed the text (around lines 158) to better explain the procedure.

3) Apparently, initiation modelling has been simplified ignoring the formation of S30-IF1 and S30-IF2 - why?

RESPONSE:
30S: can bind to IF1 and IF2, but these are subsequent after formation of 30S: IF3. This was justified by Milon et al. (see Nature structural & molecular biology. 2012;19(6):609) where the predominant pathway for 30S:PIC formation was through the intermediates 30S:IF3 → 30S:IF3IF2 → 30S:PIC. This is been noted in the supporting information which discusses this paper. Formation of the minor 30S:IF2 and 30S:IF1 species can also be incorporated into the model, with only minor changes to the code (~ 5-10 lines of code).

4) In general: Considering the 'artificial' transcriptome for simulating translation is certainly important and highly attractive. Nevertheless, some findings were already published by other others who focused on single genes alone. Examples are: Niess et al. already discussed impacts on tRNA shortage and stochasticity on translation efficiency, Vieira et al implemented stochastic translational elements, Arnold et al. already published a very complex ODE model in 2005. The author is invited to check further details in the following list:

Arnold, S., Siemann-Herzberg, M., Schmid, J., and Reuss, M. (2005). Model-based Inference of Gene Expression Dynamics from Sequence Information. In Biotechnology for the Future, (Springer, Berlin, Heidelberg), pp. 89–179

Mehra, A., and Hatzimanikatis, V. (2006). An Algorithmic Framework for Genome-Wide Modeling and Analysis of Translation Networks. Biophysical Journal 90, 1136–1146.

Nieß, A., Failmezger, J., Kuschel, M., Siemann-Herzberg, M., and Takors, R. (2017). Experimentally Validated Model Enables Debottlenecking of in Vitro Protein Synthesis and Identifies a Control Shift under in Vivo Conditions. ACS Synth. Biol. 6, 1913–1921.

Nieß,A., Siemann-Herzberg,M. and Takors,R. (2019) Protein production in Escherichia coli is guided by the trade-off between intracellular substrate availability and energy cost. Microbial Cell Factories, 18

Shaw, L.B., Zia, R.K.P., and Lee, K.H. (2003). Totally asymmetric exclusion process with extended objects: A model for protein synthesis. Phys. Rev. E 68, 021910.

Vieira, J.P., Racle, J., and Hatzimanikatis, V. (2016). Analysis of Translation Elongation Dynamics in the Context of an Escherichia coli Cell. Biophys. J. 110, 2120–2131.

Zouridis, H., and Hatzimanikatis, V. (2007). A Model for Protein Translation: Polysome Self-Organization Leads to Maximum Protein Synthesis Rates. Biophysical Journal 92, 717–730.

Zouridis, H., and Hatzimanikatis, V. (2008). Effects of Codon Distributions and tRNA Competition on Protein Translation. Biophysical Journal 95, 1018–1033.

RESPONSE:

Thank you for these references. I have looked them over and added several to the discussion section to provide context to readers on the predictions made by the various models. While I agree that some findings were published before by other groups, in particular on the consequences of codon bias and tRNA abundances, these works reflected on the competition of tRNAs for the A-site in the ribosome. There is a key difference between the work here and these previous works. Specifically, in addiction to competition of tRNAs for the A-site, this work also takes into account the consequences of recharging of elongation factors Ef-Tu and re-formation of ternary complex after consumption by elongating ribosomes and the competition of charge tRNAs in this stage. Consequences of these reactions are highlighted in the two points a and b below.

(a) The movement of the ribosome on the mRNA, and the consumption of tRNAs, are not the only processes related to the ribosome that are occurring in the cell. In addition, there are many subprocess that need to occur in order for the ribosome to have sufficient substrate to synthesise protein. Examples of these subprocesses are (1) the re-charging of ternary complex, and (2) exchange of GTP/GDP on GTPases such as Ef-Tu, Ef-G, and RF3. Although the stochastic models that are highlighted by the referee do simulate ribosome kinetics taking into account tRNA abundances, they do not include all of these subprocess. In contrast, all of the kinetic steps of elongation, and its dependence on Ef-G concentration and ternary complex concentrations, are taken into account in the model reported here along with their recharging reactions. For example, although the model in Vieira (2016 BioPhys J, 110, 2120) takes into account tRNA concentrations and their competition for the A-site in the ribosome, recharging reactions for the tRNAs and reformation into TC is modelled as instantaneous. The result of neglecting the recharging of Ef-Tu and subsequent formation of ternary complex is that the relative concentrations of the tRNAs will be approximately constant over the simulations. This allows these models to look at competition between tRNAs in TC during the elongation cycle, but it neglects the behind the scenes competition of newly aminoacylated tRNAs for newly recharged Ef-Tu to form TC. In contrast, taking formation of ternary complex, and the re-charging of Ef-Tu by Ef-Ts into account as I have done here, the relative concentrations of tRNA **in ternary complex** is dynamic and can shift over time as a result of the codon bias of the transcriptome (see Figure 4 in the manuscript). This is because as tRNAs are consumed, they must be recharged and then rebound to new Ef-Tu:GTP to form new ternary complex resulting in a competition between charged tRNAs for formation of ternary complex. Underused tRNAs will have an advantage over overused ones in formation of TC, unless consumption of tRNAs matches codon bias of the transcriptome. This can result in starvation for certain amino acids, even though there is an abundance of the amino acid and charged tRNAs in the cell, due to a lack of these tRNAs in TC. As a result, the overall effects on average translational speed from differences between the codon bias of the transcriptome and the amounts of tRNAs needed to decode the codons are much stronger than previously appreciated. This was one of the main results of the model and

demonstrates how removal of certain reactions can have dramatic effect on the results.

This manuscript reports on these different and new results, specifically the strong impact that Ef-Tu recharging reactions have on the translation process and its implications on the regulation of tRNA concentration in the cell. To demonstrate the implications of removing the Ef-Tu recharging reactions (and subsequent formation of TC) on the translational process, I have removed these reactions from my model and allowed consumed tRNAs to immediately reform into TC after release from the ribosome. This is equivalent to what was done by Vieira (2016 BioPhys J, 110, 2120). I have re-done the calculations on T10a (which has codon bias following E coli proteome) and T10 (which has an artificial codon bias following the tRNA propensities of Dong.) Both simulations were run using tRNA abundances from Dong. The result is that, once the Ef-Tu recharging reactions are removed, translation on the T10a is now much more efficient (with average 17.7 aa/sec translation speed) and similar to T10 (with average 18.4 aa/sec). When Ef-Tu recharging reactions are included, the T10a has very poor translational efficiency (average 8.1 aa/sec translation speed over the transcriptome) compared with T10 (average of 18.1 aa/sec). Thus, when Ef-Tu recharging reactions are taken into account, tRNA abundances must match their usage in the transcriptome closely to avoid shifts in the relative concentrations of tRNA in TC. This is why I have used the artificial transcriptome T10, as there is not a precise measurement of tRNA abundances. The easiest “fix” to demonstrate that mismatch was the source of the slow translation when Ef-Tu recharging is accounted for was to alter the codon bias of T10 to match tRNA abundances in Dong.

(b) There is also a second observation which has not been observed before which relates to emergent behaviour of the translational system. It is well known experimentally that in bacteria, the translation rate (number of amino acids incorporated by the ribosome per second) increases from around 15 aa/sec at a growth rate of 0.7 doublings per hour to 22 aa/sec at a growth rate of 2.5 doublings per hour. This feature emerges from the model on its own accord. Specifically, it is not enforced at all in the parameters of the model and manifests simply from the complex interactions between all of the components in the system. To my knowledge, there is no comprehensive model of translation which predicts/observes this feature. Indeed, the study by Vieira (2016 BioPhys J, 110, 2120) showed that there was no increase, contrary to experiment. This was due to the fact that the effects of Ef-Tu recharging and effects from recycling factor concentrations were not included. Once both of these are accounted for, as is done here, the increase is observed in the model.
