# Supplementary material for: A stochastic model for simulating ribosome kinetics in vivo
Source: PLoS Comput Biol. 2020 Feb 12;16(2):e1007618. doi: 10.1371/journal.pcbi.1007618 (PMC7015319; doi:10.1371/journal.pcbi.1007618)
Supplement: Attachment — Submitted filename: response2.pdf [file pcbi.1007618.s003.pdf]

Reviewer #1: I thank Dr Dykeman for the detailed response to my comments.

I am generally satisfied with the response to the first two comments. With respect to the "novelty" aspect, my evaluation was too focused on the translation process itself and I had not considered all the additional reactions included in the model, so upon reflection I agree with Dr Dykeman's argument in this respect. The details of the modified Gillespie algorithm had escaped me but are now clearer and easier to understand for non-experts such as myself.

I do however have continuing concerns about the decision to base the majority of the study on a non-physiological transcriptome. Expressed in a slightly snide way to drive home the point, the message is essentially that "because my model does not fit well with experimental data, I make up a dataset that fits the model better and base my study on that". I thought about this for quite a while because I would like to be fair to the study and its author, but in the end this is not an approach I wish to condone for the following reasons:

1) The assumption that it is the experimental data that are are inaccurate, rather than the model, seems arbitrary in the first instance.

2) Even if the model is correct and some of the experimental data inaccurate, the transcriptome replacement strategy is underpinned by the very specific but arbitrary notion that it must be the tRNA data that are wrong. Why can it not be the target amino acids per second data that are wrong, or the recharging data (nb "recharging" in the manuscript should actually be "nucleotide exchange" – recharging traditionally refers to tRNA synthetase reactions). It is clearly acknowledged that altering elongation factor re-charging can produce high elongation rate with the actual E. coli transcriptome...

3) One of the motivations for replacing the transcriptome is the assumption that tRNAs and codon usage should match perfectly (most directly expressed in lines 283-287). This seems to take that literature observation that there is some correlation between codon usage and tRNA content and interpret it very strongly.

4) Overall, I think this study could be re-framed as one that highlights apparent mismatches in experimental data revealed by the modelling, and carefully discusses the potential implications. However, I do not think the current framing where an (in my view) uncertain work-around to the mismatch is provided and this is then used to make strong statements about biological implications works very well.

RESPONSE: The reviewer notes in several points above that there could be alternative explanations as to origin of the slow translation rates in the model when using experimental measured tRNA abundances from Dong et al. and WT E coli mRNAs. For instance, it is possible that the model is missing additional chemical reactions or requires alterations to other parts of the model instead of tRNA abundances (such as kinetic rates) which would fix the issues around translation rates and tRNA abundances. I completely agree and note that it was a bit unfair of me to make the discussion in the manuscript strongly imply that the only solution to the issue was via a precise matching between codon usage in the transcriptome and tRNAs. Indeed, alternative plausible explanations should also be addressed and considered in the manuscript. It certainly was not my intention to imply that the experiments done by Dong and colleagues were somehow inferior to modelling. On the contrary, I have much respect for their work and without their important insights and very nice experimental work I would have been lost during the fitting

of my model. As noted in the manuscript, when you add in more of the reactions that are taking place in the cell, suddenly the translational process is very sensitive to the codon composition of the transcriptome. This could be a consequence of the high translation rate of E. coli and in other cells, such as yeast where the avg. translational speed is slower at 5-8 aa.sec, the translational machinery may be able to better cope with differences between codon bias and tRNA abundance. I can see at least five ways in which the translational speed discrepancy for the prokaryotic system can be alternatively explained.

1) The abundance of tRNAs relative to codon usage needs to be optimized in some way.
2) The kinetic rates of translation elongation process (or other rates such as those for aa-tRNA synthase) need adjustment
3) Additional reactions, potentially unknown experimentally, need to be accounted for.
4) There is no discrepancy, and translational speed is actually slower then what was previously measured experimentally.
5) The abundance of tRNA and translational kinetics need adjusted together in some way.

I believe it is likely the last one that is the actual way forward as Rudorf et al previously altered the kinetic rates of the elongation process to fit to the Dong tRNA profile. This suggests that a simultaneous optimization of both tRNA and the elongation kinetic rates is needed, possibly combined with optimization of amino-acyl tRNA synthase reactions. This is a complicated and quite difficult process, hence why I have resorted to the non-physiological transcriptome. However, I take your point that this Is a slightly unfair “fudge” to make the model reproduce experiment. However, I do have good evidence from the modelling that altering the tRNA abundances so that they match codon distributions from WT E. Coli does address most of the issues with slow translation. I have now added a section on modelling with WT-E coli transcriptome and the issues with reproducing experimental observations. Using WT-E coli transcriptomes with the Dong tRNA profile, translation at the mu=1.0 growth rate is ~ 4 aa/sec. But if one uses an altered tRNA abundance profile which matches with the codon frequency, this increases to 15 aa/sec. This is slightly under the 18 aa/sec expected, so additional alterations to either the tRNA abundances or other kinetic rates is needed. (Probably an optimization of elongation rates and tRNA abundances in tandem, while minimizing the differences between experimental measurements of the tRNAs and kinetic elongation rates as done in Rudorf – PLOS comp bio 10, e1003909)

I believe that the revision now reports the model in a fair light and, combined with the use of the non-physiological transcriptome, highlights interesting unanswered questions on the topic of how tRNA recycling is regulated in the cell to insure efficient translation. In addition to reporting on a technique for simulating the translation process from a more holistic view, the other purpose of the paper is to stimulate both theoretical and experimental work around this question and the use of the non-physiological transcriptome helps aid this debate.

The very next paper I have planned is a systematic examination of tRNA concentrations, elongation kinetic rates and aminoacyl-tRNA synthase reactions to pinpoint the sensitivity that each have on the translation process in the presence of WT E. coli transcriptomes. This is an in-depth analysis and I have been working on finishing this shortly. But because my model can incorporate these additional reactions using an exact Gillespie firing individual reactions one at a time, it is capable of probing such a sensitivity analysis in the context of a full transcriptome. This is one of the reasons I believe that the model and work that I present here will be important to the community.

Reviewer #2: Review of PCOMPBIOL-D-19-01692R1

My original comment was fully addressed and I still believe that this manuscript deserves publication.

However, I now disagree with some parts of the revised manuscript and these issues should be addressed prior to publication.
In my opinion, the great advancement of the presented work is the following:
by integration of many different biomolecular processes that govern protein biosynthesis into a “holistic” model, many important features of translation (growth rate dependence of translation rates, sensitivity to codon/tRNA composition, usage of the machinery) are shown to be inherent to the system and can be explained without any further assumptions. Neglecting some parts of the system, such as ternary complex formation, leads to a loss of these characteristic features.

I agree that it is important to stress this point but while doing so the author must be careful to not make the impression as if some of the _individual features_ have not been studied before. In particular, in the new text the author writes
- “While previous stocahstic models have examined competiton between tRNAs in TC for the A-site in elongating ribosomes [2–4], they have not considered the recharging reactions, specifially GTP/GDP exchange on Ef-Tu and formation of TC” (l 324ff)
- “…the model I report on here takes into account effects from the additional competition that occurs between aminoacyl-tRNAs for Ef-Tu:GTP during formation of free TC after recharging of tRNA by aminoacyl-TRNA synthase” (l 422ff)
- “… the effects on the translational rate from the competition between aminoacyl-tRNAs for formation of free TC is much more dramatic” (l 426f)

It is not true that the great influence of ternary complex formation on the translation rate has not been described before. In contrast, that the competition for EF-Tu during TC formation has a tremendous effect on the overall rate of protein synthesis was recently studied and described in great detail (PMID 31369559). Note that neglecting the GDP/GTP exchange does not cause a fundamental difference between the published and your model, because including the GDP/GTP exchange basically reduces the effective concentration of EF-Tu available for ternary complex formation. The mentioned paper demonstrates that such a reduction (independent of its origin) strongly affects protein synthesis.
- “Thus, situations where the relative abundances of tRNAs do not match their demand implied by the codon bias of the transcriptome will result in shifts in the amount of tRNA available in free TC” (l 330ff)
- “This observation implies that tRNA abundances must closely match the levels of demand placed on them as a result of the codon bias in the transcriptome for efficient translation to occur” (l 428ff)
In the paper mentioned above (PMID 31369559) it was shown in detail that a disbalance between codon usage and tRNA abundance is the driving force behind the sensitive dependence of translation on TC formation (i.e., EF-Tu availability). That this causes “shifts in the amount of tRNA available in free TC” was also already demonstrated previously (PMID 26270805).
- “… removing the reactions involved in Ef-Ts mediated GDP/GTP exchange on Ef-Tu, a feature that is often neglected in translational models, resulted in similar average translational speeds for T10a and T10 transcriptomes, despite the difference between the demand for tRNA species implied by the codon usage in T10a and the tRNA abundances present”(l 430ff)

Again: That (effectively) increasing the amount of EF-Tu available for TC formation (which is exactly what happens when GDP/GTP exchange is ignored) relieves the sensitivity of the speed of translation for disbalances in the tRNA/codon composition was demonstrated before (PMID 31369559). The observation made by the author is thus in full agreement with the previously published model and the statement is important to make because in most translation models the strong impact of the availability of EF-Tu for TC formation is indeed ignored. But again, the author should mention that he is not the first/only to make this observation and must put his results in the context of previous publications.

Side note: This implies that the strong dependency of the results on the chosen transcriptome is "caused" by the chosen value for the EF-Tu concentration. You could check if the differences between T10 and T10a still remain when you increase the EF-Tu by e.g. 10, 20 or 50 % (but keeping the GDP/GTP exchange reaction). At some point the differences should vanish.
In this context one could also discuss that EF-Tu has many functions in bacteria in addition to its main role as elongation factor. That means that other cell processes sequester EF-Tu, thus reduce the amount of EF-Tu available for TC formation and increase the need for a well-balanced tRNA/codon composition.

RESPONSE: The reviewer makes several very good points about the reading of the text and referencing of prior work. One work in particular (PMID 31369559) I was genuinely unaware of. I agree and apologize for the way that the text was written since in its previous form it can be mis-interpreted. So, I have adjusted the areas noted by the referee and added the additional reference (PMID 31369559). Hopefully the text is now a fairer assessment of the literature, but if not please let me know.

Regarding the comment on the Ef-Tu concentration. You are correct. If I increase Ef-Tu concentration (along with Ef-Ts to allow GTP/GDP exchange) the elongation rate increases from 8 aa/sec for the T10a transcriptome to 12 aa/sec, 14 aa/sec and 15 aa/sec when Ef-Tu is increased by 25,50, and 75 %. This agrees with the observations from (PMID 31369559), and I have added some text discussing this.
